# Arabidopsis protein disulfide isomerase-8 is a type I endoplasmic reticulum transmembrane protein with thiol-disulfide oxidase activity

**DOI:** 10.1186/s12870-016-0869-2

**Published:** 2016-08-22

**Authors:** Christen Y. L. Yuen, Roger Shek, Byung-Ho Kang, Kristie Matsumoto, Eun Ju Cho, David A. Christopher

**Affiliations:** 1 Department of Molecular Biosciences and Bioengineering, University of Hawaii, 1955 East-West Rd., Ag. Science Rm 218, Honolulu, HI 96822 USA; 2 The Chinese University of Hong Kong, School of Life Sciences, Shatin, Hong Kong, SAR China

**Keywords:** Endoplasmic reticulum, Transmembrane, Protein disulfide isomerase, Protein folding

## Abstract

**Background:**

In eukaryotes, classical protein disulfide isomerases (PDIs) facilitate the oxidative folding of nascent secretory proteins in the endoplasmic reticulum by catalyzing the formation, breakage, and rearrangement of disulfide bonds. Terrestrial plants encode six structurally distinct subfamilies of PDIs. The novel PDI-B subfamily is unique to terrestrial plants, and in Arabidopsis is represented by a single member, PDI8. Unlike classical PDIs, which lack transmembrane domains (TMDs), PDI8 is unique in that it has a C-terminal TMD and a single N-terminal thioredoxin domain (instead of two). No PDI8 isoforms have been experimentally characterized to date. Here we describe the characterization of the membrane orientation, expression, sub-cellular localization, and biochemical function of this novel member of the PDI family.

**Results:**

Histochemical staining of plants harboring a *PDI8* promoter:β-glucuronidase (GUS) fusion revealed that the *PDI8* promoter is highly active in young, expanding leaves, the guard cells of cotyledons, and in the vasculature of several organs, including roots, leaves, cotyledons, and flowers. Immunoelectron microscopy studies using a PDI8-specific antibody on root and shoot apical cells revealed that PDI8 localizes to the endoplasmic reticulum (ER). Transient expression of two PDI8 fusions to green fluorescent protein (spGFP-PDI8 and PDI8-GFP-KKED) in leaf mesophyll protoplasts also resulted in labeling of the ER. Protease-protection immunoblot analysis indicated that PDI8 is a type I membrane protein, with its catalytic domain facing the ER lumen. The lumenal portion of PDI8 was able to functionally complement the loss of the prokaryotic protein foldase, disulfide oxidase (DsbA), as demonstrated by the reconstitution of periplasmic alkaline phosphatase in *Escherichia coli*.

**Conclusion:**

The results indicate that PDI8 is a type I transmembrane protein with its catalytic domain facing the lumen of the ER and functions in the oxidation of cysteines to produce disulfide bonds. It likely plays a role in folding newly-synthesized secretory proteins as they translocate across the ER membrane into the lumen. These foundational results open the door to identifying the substrates of PDI8 to enable a more thorough understanding of its function in plants.

**Electronic supplementary material:**

The online version of this article (doi:10.1186/s12870-016-0869-2) contains supplementary material, which is available to authorized users.

## Background

Many proteins that transit through the secretory pathway require disulfide bonds to stabilize their native functional conformation. Disulfide bond formation in secretory proteins primarily occurs within the endoplasmic reticulum (ER), and is mediated by members of the protein disulfide isomerase (PDI) family. The classical PDI (represented by PDIA1 in mammals, and PDI1 in *Saccharomyces cerevisiae*) is a versatile enzyme capable of catalyzing the oxidation, reduction, or isomerization of disulfide bonds in a wide range of substrate proteins *in vitro* [5], and can also assist in protein folding as a molecular chaperone [21, 32]. The classical PDI structure consists of four modular domains in the arrangement **a-b-b’-a’**, where **a** and **a’** are catalytic domains sharing homology to thioredoxin [9]. The catalytic domains contain a redox-active vicinal dithiol comprised of two cysteines separated by two amino acids (CxxC). In contrast, the **b** and **b’** domains lack sequence homology to thioredoxin, but possess the βαβαβαββα thioredoxin structural fold [16], with the **b’** domain serving as the principle binding site for misfolded proteins [15]. In the case of the pancreas-specific human PDI homolog, PDIA2, the **b-b’** region is associated with chaperone activity [11].

Although PDIs with the **a-b-b’-a’** structure are conserved across animals, plants and yeasts, there is a diverse assortment of PDI-like proteins that deviate from this arrangement. Terrestrial plants encode six structurally divergent PDI subfamilies, designated as A, B, C, L, M and S [26]. The 14 total PDIs of the model dicot, *Arabidopsis thaliana*, comprise six isoforms of PDI-L, three isoforms of PDI-C, two isoforms of PDI-M, and a single isoform each of PDI-A, PDI-B, and PDI-S. While the functions of most Arabidopsis PDI proteins have not been elucidated, there is growing evidence that several PDIs have evolved to take on distinct roles in plant growth and development. Members of the PDI-L subfamily (PDI1, PDI2, PDI3, PDI4, PDI5, and PDI6) share the **a-b-b’-a’** arrangement of classical PDIs and primarily localize to the ER [37], although PDI5 is also present in protein storage vacuoles [2], PDI6 in chloroplasts [34], and PDI2 in both vacuoles and the nucleus [6, 24]. Whereas PDI5 influences embryo development by chaperoning and inhibiting cysteine (Cys) proteases involved in programmed cell death [2], its sister paralog PDI6 was implicated as a modulator of photoinhibition [34]. PDI2 interacts with both the ER resident chaperone, BiP, and the nuclear transcription factor, MEE8 (maternal effect embryo arrest 8), and is highly expressed in seeds, suggesting an involvement in embryo/seed development [6].

PDI-M and PDI-S isoforms contain two catalytic **a**-type domains, but without the intervening redox-inactive **b**-type domains found in PDI-L isoforms [20]. PDI-M isoforms have an **a**^**0**^**-a-b** domain arrangement and are co-orthologs of mammalian PDIA6 [26], while PDI-S isoforms have an **a**^**0**^**-a-D** arrangement, where **D** represents a conserved all α-helical domain of unknown function [10]. Arabidopsis isoforms of PDI-M (PDI9 and PDI10) and PDI-S (PDI11) both localize to the ER [31, 37], with the PDI-M isoforms accumulating within microdomains of the ER known as ER bodies [37]. In Arabidopsis, the expression of truncated versions of PDI11 disrupts both pollen tube guidance and embryo sac development [31].

Two striking examples of PDIs that deviate from the classical **a-b-b’-a’** domain arrangement are the PDI-B and PDI-C sub-families. Unlike the majority of the PDI family, PDI-B and PDI-C are predicted to contain one or two transmembrane domains (TMDs), respectively [20, 26]. Although PDI-B and PDI-C are both putative transmembrane PDIs, they contain unique structural features that set them apart from each other. PDI-C isoforms possess a single catalytic **a** domain, flanked on both ends by sequences homologous to yeast Erv41p and Erv46p [38], which have recently been implicated as cargo receptors for the retrieval of ER proteins lacking the traditional yeast ER retention signal, HDEL [27]. By contrast, PDI-B isoforms possess an **a-b-b’** domain arrangement that is reminiscent of classical PDI structure, but PDI-B isoforms lack a second catalytic (**a’**) domain and instead possess a C-terminal TMD [20, 26]. PDI-B and PDI-C isoforms have not been experimentally characterized to date. The PDI-B subfamily is represented by a single member in Arabidopsis, PDI8 (Arabidopsis Genome Identifier At1g35620). Here we describe the characterization of the membrane orientation, expression, sub-cellular localization, and biochemical function of this novel member of the PDI family.

## Results

### Domain architecture and sequence characteristics of PDI8

**Fig. 1:**
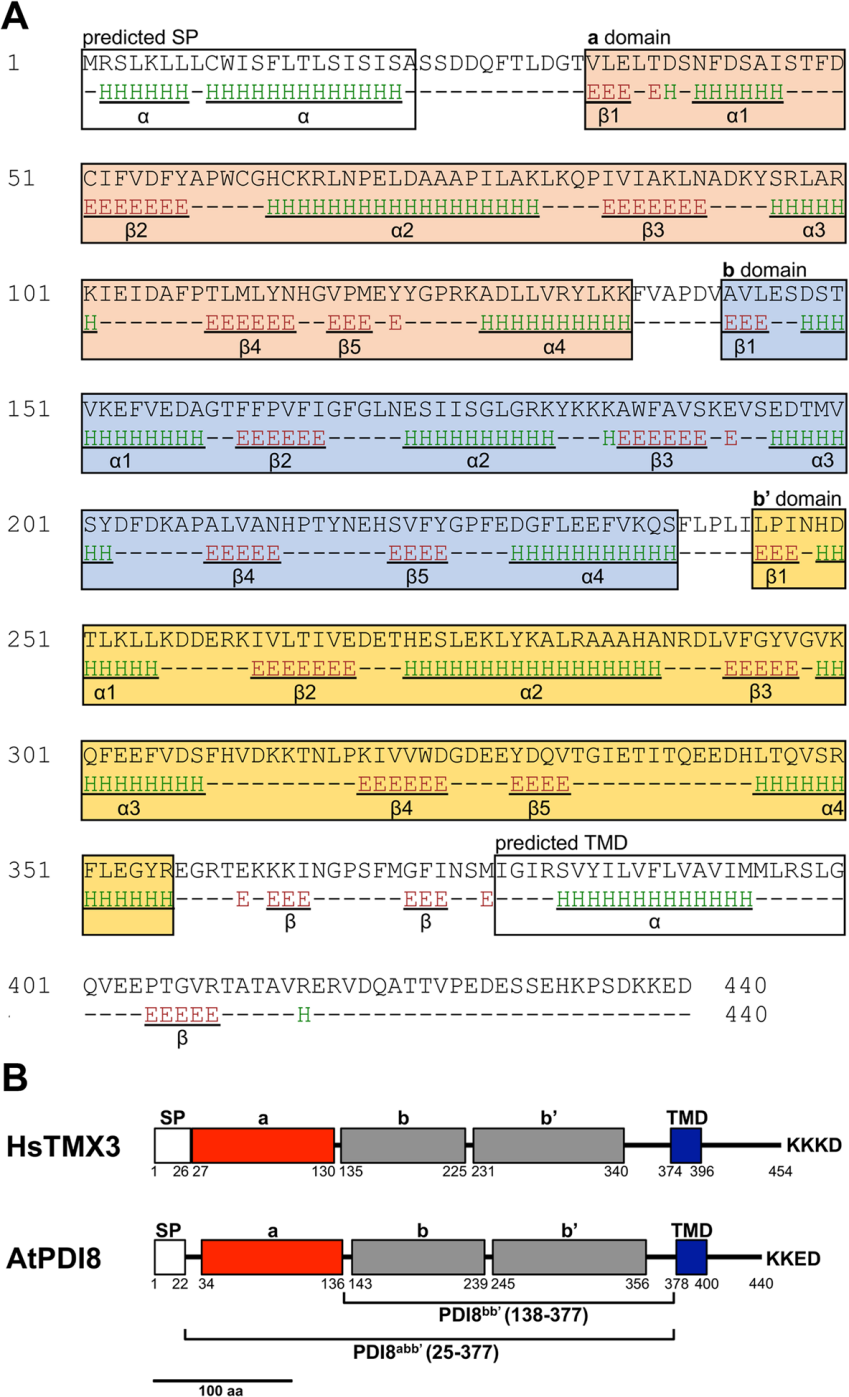
Domain arrangement of PDI8. **a** The secondary structure of PDI8. Positions of α-helices (E) and β-strands (H) are based on prediction by SPIDER2. The thioredoxin-fold domains (***a***, ***b*** and ***b’***), and predicted signal peptide (SP) and TMD of PDI8 are boxed. Each thioredoxin fold consists of 5 β-strands and 4 α-helices (underlined), in the arrangement β_1_-α_1_-β_2_-α_2_-β_3_-α_3_-β_4_-β_5_-α_4_. **b** Comparison of the domain organizations of *Homo sapiens* TMX3 and Arabidopsis PDI8, showing the relative positions of the SP, TMD, and domains ***a***, ***b*** and ***b’***. PDI8^abb’^ and PDI8^bb’^ represent truncated forms of PDI8 used in this study

The Arabidopsis *PDI8* gene contains five exons and encodes a deduced polypeptide of 440 amino acids [20]. The first 22 amino acids of the deduced PDI8 sequence are predicted by SignalP-4.1 to serve as a cleavable signal peptide (mean S value = 0.936), with the resulting mature PDI8 protein having a calculated molecular weight of 47.4 kDa and a theoretical pI of 5.01. PDI8 is predicted by TMHMM v. 2.0 to contain a single TMD, spanning residues 378-400 of the PDI8 preprotein sequence. Secondary structure prediction of the PDI8 preprotein by SPIDER2 revealed an alternating pattern of α-helices and β-strands, including three intervals with the thioredoxin structural fold, βαβαβαββα (Fig. 1a). Protein domains belonging to the thioredoxin fold class are identified on the basis of their secondary structural elements, rather than actual sequence homology to the cytoplasmic redox protein, thioredoxin [4]. Despite their predicted structural resemblance to thioredoxin, the three thioredoxin-fold domains of PDI8 do not share significant sequence homology to each other, and only the first domain (domain **a** in Fig. 1a) shares homology to canonical thioredoxin proteins.

By convention, PDI redox-active thioredoxin-fold domains are referred to as **a** domains, while redox-inactive thioredoxin-fold domains are termed **b** domains [3]. The N-terminal-most thioredoxin-fold domain of PDI8 is an **a**-type domain sharing 42 % and 35 % sequence identity with the **a** and **a’** domains of human PDI, respectively, and contains the CGHC redox active site motif found in the **a** and **a’** domains of the classical PDIs from human and yeast. The other two thioredoxin-fold domains of PDI8 do not contain any potentially redox-active Cys residues, and were thus designated as **b**-type domains (**b**, **b’)**. BLAST searches of the Arabidopsis TAIR10 protein database indicated that the **b** and **b’** domains of PDI8 do not share significant homology to other proteins from Arabidopsis, including the **b** and **b’** domains of other members of the PDI family. Furthermore, although PDI8 shares a similar domain arrangement to human thioredoxin-related membrane protein 3 (TMX3; Fig. 1b), no homology was found at the amino acid level between the **b**-type domains of PDI8 and TMX3 in pairwise sequence similarity comparisons using the BLAST algorithm.

Consistent with prior genomic analyses of the plant PDI family [8, 20, 26], we identified at least one ortholog of PDI8 among all monocot and dicot species surveyed, as well as among the model bryophyte *Physcomitrella patens* and the lycophyte *Selaginella moellendorffii*, while no PDI8 ortholog was evident among the genomes of representative chlorophyte green algae species (Table 1). BLAST searches using the unique **bb’** region of Arabidopsis PDI8 also failed to identify any orthologs of PDI8 among non-plant species, indicating that the PDI-B subfamily is specific to plants. Nearly all monocot and dicot PDI8 orthologs possess the classical PDI dithiol active site sequence, CGHC, although one of the two PDI8 orthologs from *Populus trichocarpa* contains the non-classical variant CTHC. Only non-classical variants of the CxxC motif were present in the PDI8 orthologs from *Physcomitrella* (CKHC, CGFC) and *Selaginella* (CSHC). The C-terminus of Arabidopsis PDI8 ends with the sequence KKED [20], which resembles the KKxx or xKxx tetrapeptide signal for ER retrieval of transmembrane proteins via COPI-coated vesicles. Comparison of the C-termini of PDI8 orthologs revealed that all dicot orthologs and the two orthologs from *Physcomitrella* shared the C-terminal motif, xKxD, while monocot PDI8 orthologs possessed the C-terminal motif xHx(E/D).

**Table 1:** Representation of the PDI-B subfamily in plants

	Species	No. of Genes	CxxC Motif	C-terminal Tetrapeptide
Chlorophytes	*Chlamydomonas reinhardtii*	0	–	–
	*Coccomyxa subellipsoidea *	0	–	–
	*C-169 Volvox carteri*	0	–	–
Bryophytes	*Physcomitrella patens*	2	CKHC	NKED
			CGFC	KKED
Lycophytes	*Selaginella moellendorffii*	1	CSHC	AARH
Monocots	*Brachypodium distachyon*	2	CGHC	IHDE
	*Oryza sativa*	2	CGHC	AHQE
			CGHC	AHEE
			CGHC	AHED
	*Sorghum bicolor*	2	CGHC	IHEE
			CGHC	AHED
	*Zea mays*	3	CGHC	IHEE
			CGHC	IHEE
			CGHC	AHED
Dicots	*Arabidopsis thaliana*	1	CGHC	KKED
	*Capsella rubella*	1	CGHC	KKED
	*Eutrema salsugineum*	1	CGHC	DKED
	*Glycine max*	1	CGHC	DKED
	*Medicago truncatula*	1	CGHC	DKED
	*Phaseolus vulgaris*	1	CGHC	DKED
	*Populus tricocarpa*	2	CGHC	DKQD
			CTHC	DKQD
	*Prunus persica*	1	CGHC	EKED
	*Solanum lycopersicum*	2	CGHC	EKID
			CGHC	DKED
	*Solanum tuberosum*	2	CGHC	DKID
			CGHC	DKED
	*Theobroma cacao*	2	CGHC	KKED
			CGHC	EKED
	*Vitis vinifera*	1	CGHC	DKED

### *PDI8* promoter expression analysis using the GUS reporter system

**Fig. 2:**
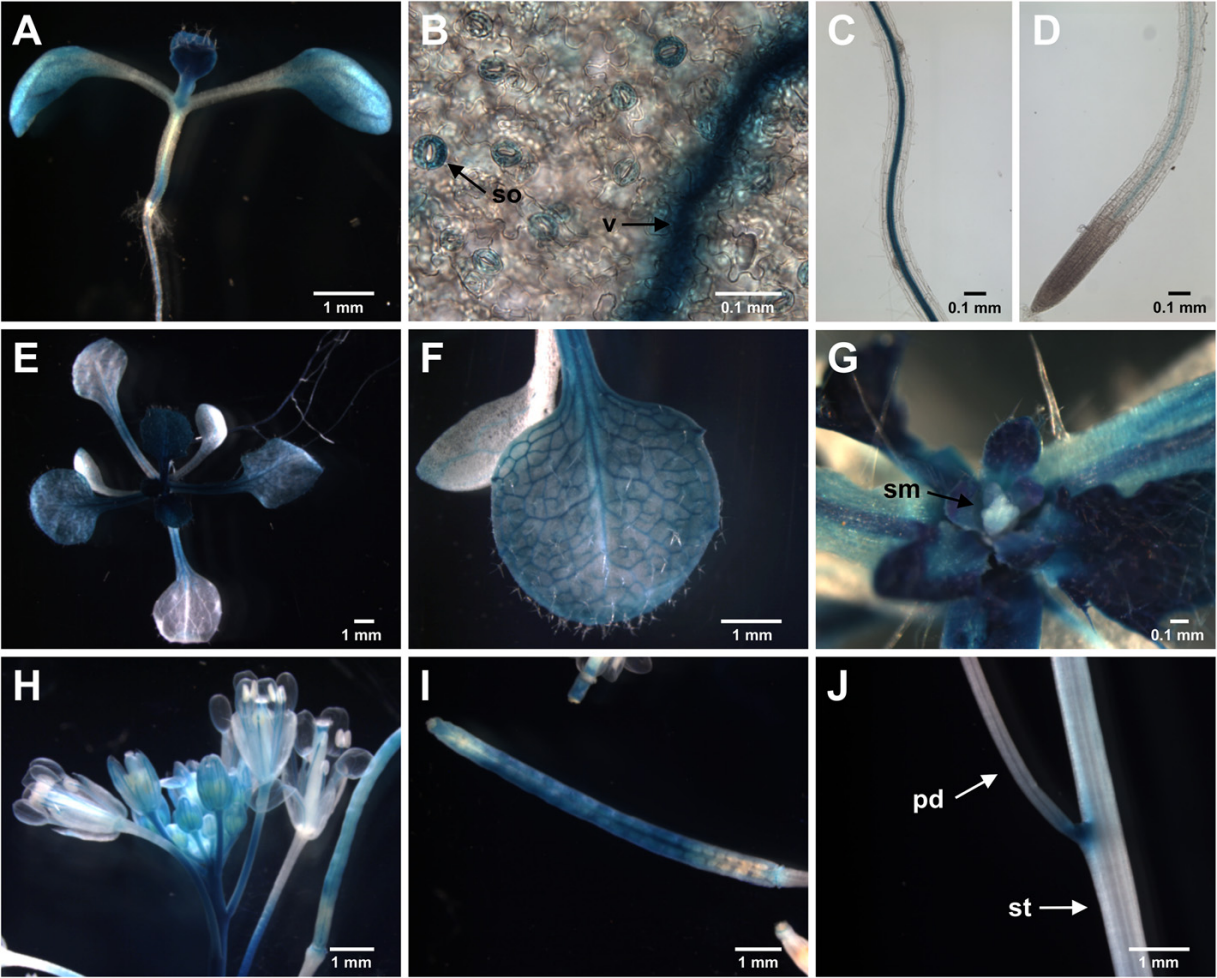
Expression pattern of the *PDI8*_*pro*_ *:GUS* reporter construct in seedlings and flowering plants. GUS staining pattern of 7-day-old seedlings (**a**), with close-up images of a cotyledon stomata (so) and vasculature (v) (**b**), the root mature zone (**c**), and the root tip (**d**). GUS staining pattern of 14-day-old seedlings (**e**), with close-up images of an expanding leaf (**f**) and the shoot apex (**g**). In panel **g**, the emerging leaves were pulled back to expose the shoot apical meristem (sm). GUS staining pattern of 6-week-old plants in flowers (**h**), an expanding silique (**i**), and the inflorescence stem (**j**). In **j**, staining is shown at the junction between the stem (st) and the pedicel (pd) of a silique

To examine the spatial expression pattern of *PDI8 in planta*, we generated transgenic Arabidopsis plants harboring the ~2.3-kb region immediately upstream of the *PDI8* start codon (including the *PDI8* promoter and 5’ untranslated region) transcriptionally fused to the reporter gene, β-glucuronidase (GUS). A total of 11 independent transgenic lines were analyzed to establish the consensus expression pattern of the *PDI8*_*pro*_*:GUS* fusion in seedlings and flowering plants. Histological staining of 7-day-old seedlings revealed strong expression of the GUS transgene in the emerging first true leaves, cotyledons, roots, and the base of the hypocotyl (Fig. 2a). In cotyledons, GUS staining was primarily detected in the vasculature and guard cells (Fig. 2b). In roots, GUS staining was observed exclusively in the vasculature, both at the mature zone (Fig. 2c) and the root tip (Fig. 2d). The staining pattern of 14-day-old *PDI8*_*pro*_*:GUS* plants (Fig. 2e) was similar to that of 7-day-old seedlings, although GUS staining in older (expanded) leaves was primarily confined to the vasculature (Fig. 2f), whereas strong GUS staining was observed throughout younger (emerging) leaves (Fig. 2g). However, we did not observe significant GUS staining at the shoot apical meristem (Fig. 2g).

In 6-week-old reproductive-stage plants, expression of the *PDI8*_*pro*_*:GUS* transgene was detected at the style, and in the vasculature of petals, sepals and stamen filaments (Fig. 2h). Strong GUS expression was also present in pedicels, although the pedicels of older flowers exhibited decreased GUS staining near the pedicel/flower junction (Fig. 2h). We also detected significant GUS expression in siliques (Fig. 2i), and the pedicel/stem junction (Fig. 2j).

The expression pattern of *PDI8* was also examined by mining publicly-available microarray data through the Bio-Analytic Resource ePlant Browser (https://bar.utoronto.ca/eplant/; [33]). Consistent with our GUS reporter expression analyses, *PDI8* transcripts were detected across many plant tissues, including roots, leaves, flowers and siliques (Additional file 1). The highest mean expression values corresponded to expanding siliques, heart and globular-stage embryos, pedicels, 24 h imbibed seeds, and the 2^nd^ internode of the inflorescence stem, while the lowest mean expression value corresponded to mature pollen.

### PDI8 localizes primarily to the ER

**Fig. 3:**
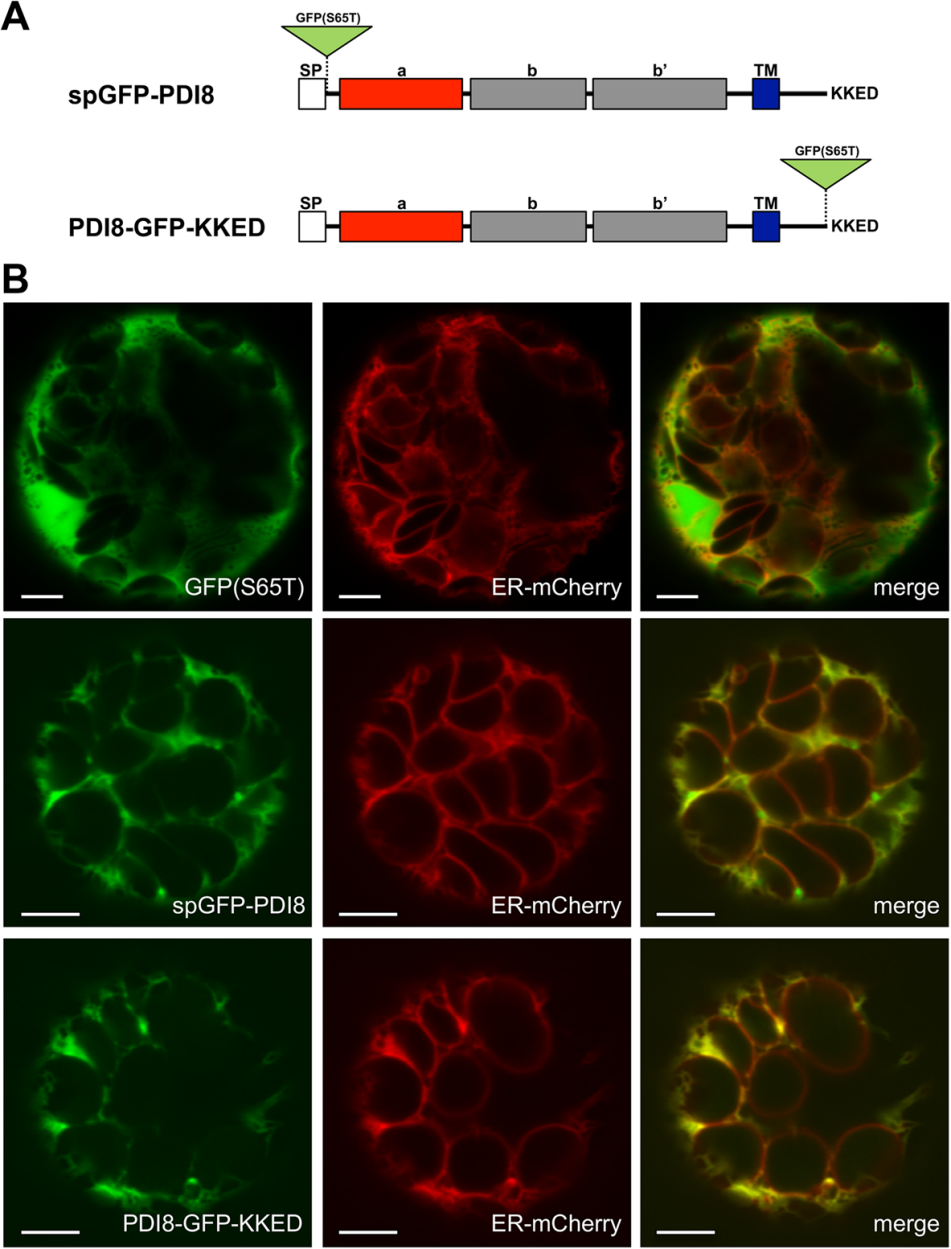
GFP fusions to PDI8 localize to the ER. **a** Position of GFP(S65T) within the fusions spGFP-PDI8 and PDI8-GFP-KKED. **b** Transient co-expression of the ER-mCherry marker with unfused GFP(S65T) (top row), the spGFP-PDI8 construct (middle row), or the PDI8-GFP-KKED construct (bottom row). GFP(S65T) signal is shown in column 1, mCherry signal in column 2, and a merge of both signal patterns in column 3. The scale bar in each panel represents 5 μm

The subcellular localization pattern of PDI8 was examined using two different approaches: 1) transient expression of a PDI8 fusion to the green fluorescent protein variant, GFP(S65T), in Arabidopsis leaf protoplasts, and 2) detection of native PDI8 in wild-type Arabidopsis ultra-thin sections by transmission immunoelectron microscopy. For the first approach, since PDI8 potentially contains both a signal peptide at its N-terminus and an ER retrieval signal at its C-terminus, we generated two constructs expressing GFP(S65T) at different positions relative to the *PDI8* open reading frame (Fig. 3a). In the spGFP-PDI8 fusion, GFP(S65T) is positioned internally between the signal peptide and mature peptide sequences of PDI8. In the PDI8-GFP-KKED, GFP(S65T) is positioned at the C-terminus of the PDI8, with the C-terminus of GFP(S65T) modified to include the putative ER retention sequence of PDI8, KKED. When transiently co-expressed in protoplasts with a marker for the ER, both the spGFP-PDI8 and PDI8-GFP-KKED fusions exhibited a subcellular distribution pattern that strongly overlapped with that of the network-like localization pattern of the ER-mCherry, whereas unfused GFP(S65T) displayed a distribution pattern that was noticeably more diffuse than the ER-mCherry marker (Fig. 3b).

To facilitate the higher-resolution subcellular localization of PDI8, a PDI8-specific polyclonal antiserum was raised in rabbits against a truncated version of PDI8 containing the **b-b’** region (PDI8^bb’^; Fig. 1b) of the protein. The reactivity and specificity of the anti-PDI8 antiserum was examined by immunoblot analysis against recombinant PDI8^bb’^, and against total protein samples extracted from 7-day-old wild-type (WT) Arabidopsis seedlings and transgenic plants expressing the *PDI8* cDNA under the strong constitutive CaMV 35S promoter in either the sense orientation (*35S*_*pro*_*:PDI8*) or antisense orientation. The anti-PDI8 antiserum strongly detected the recombinant PDI8^bb’^ protein (Additional file 2a), and a protein with a MW of ~54 kDa in both WT and *35S*_*pro*_*:PDI8* lines (Additional file 2b). The 54-kDa protein was detected very strongly in *35S*_*pro*_*:PDI8* overexpression lines relative to WT, indicating that this protein corresponds to PDI8 in plants. We did not observe any phenotype associated with either overexpression or antisense suppression of *PDI8*. However, analysis of transcript levels in the *PDI8* antisense lines by quantitative reverse transcription PCR (RT-qPCR) showed that the endogenous *PDI8* gene was only partially suppressed in these lines (40–50 %), indicating that the obtained antisense lines were not true knockouts (data not shown).

**Fig. 4:**
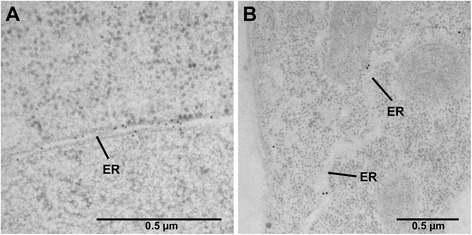
Detection of native PDI8 specifically at the ER by immunoelectron microscopy. TEM analysis was performed on sections taken from the shoot apex (**a**), and the root apex (**b**), after primary labeling with rabbit anti-PDI8 antiserum and secondary labeling with 10 or 15 nm gold-conjugated goat anti-rabbit IgG antibodies (respectively). Labeling was detected at the endoplasmic reticulum (ER)

For a high resolution analysis of the localization pattern of native PDI8, we prepared specimens for immunogold labeling from the shoot and root apices of wild-type Arabidopsis seedlings using high-pressure freezing and freeze-substitution. After sectioning the specimens, they were labeled with the anti-PDI8 antiserum, followed by secondary labeling with a gold-conjugated anti-rabbit antiserum. In shoot apical cells, prominent labeling of the ER by the anti-PDI8 antiserum was observed (Fig. 4a). This antiserum also labeled the ER in root apical cells (Fig. 4b). We did not detect significant anti-PDI8 labeling of any other sub-cellular structures. No labeling was observed using the pre-immune serum on sections from wild-type seedlings nor using the anti-PDI8 antiserum on the antisense line (Additional file 3a, b, c). Thus, the ER labeling observed using the anti-PDI8 antiserum (Fig. 4a) was specifically detecting PDI8. Sections from *35S*_*pro*_*:PDI8* overexpression lines labeled with the anti-PDI8 antiserum displayed strong labeling of the ER, indicating that the overexpression of PDI8 in plants does not lead to mislocalization of the protein (Additional file 3d).

### PDI8 is a type I integral membrane protein

**Fig. 5:**
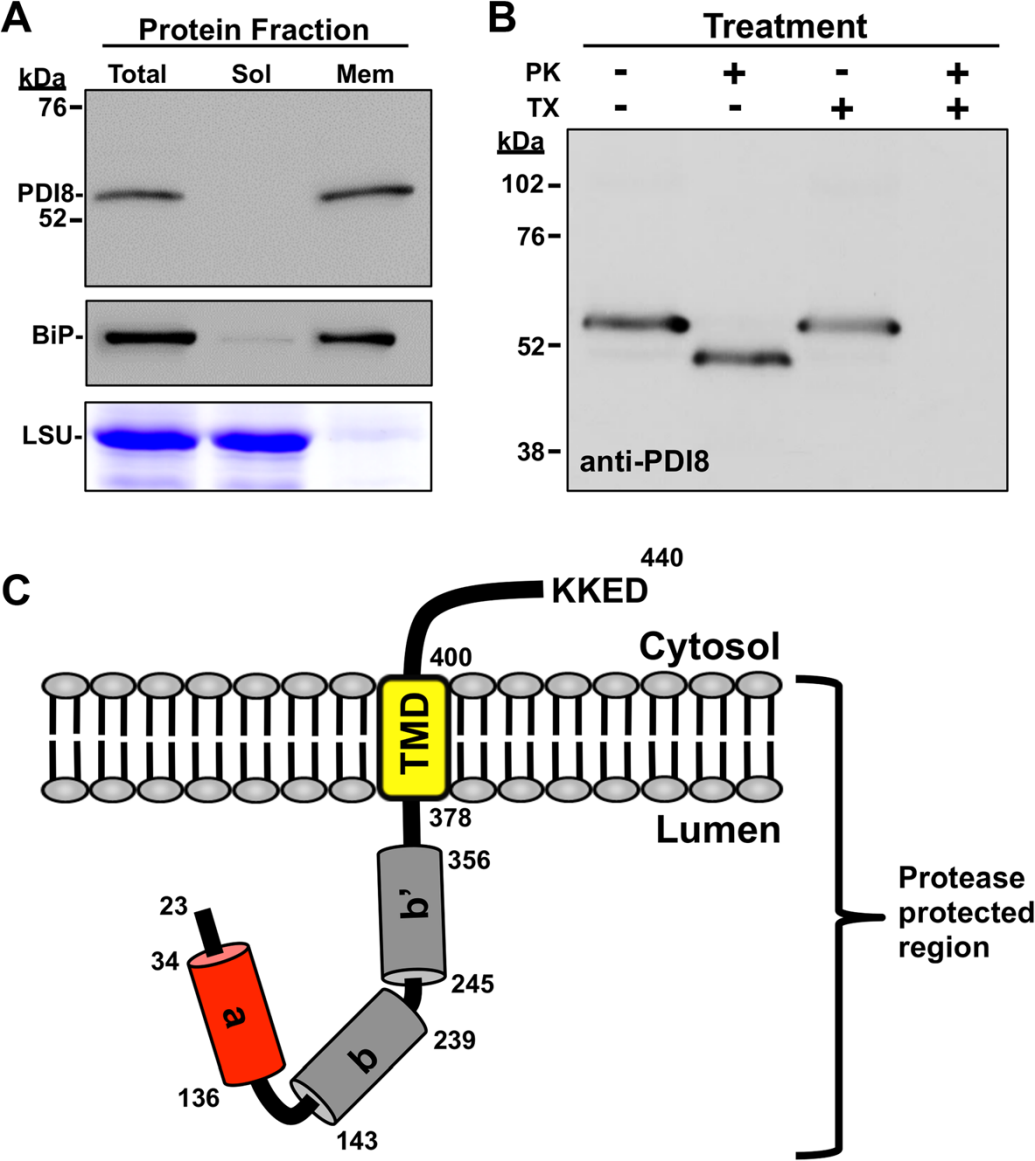
Membrane orientation of PDI8. **a** Immunoblot analyses of proteins extracted from *35S*_*pro*_ *:PDI8* plants. The *35S*_*pro*_ *:PDI8* total protein homogenate was separated into soluble (sol) and microsomal membrane (mem) fractions by centrifugation. Immunoblots were incubated with affinity-purified anti-PDI8 antiserum (upper panel). A polyclonal goat anti-BiP antibody was used as a marker for microsomes (middle panel). The large subunit of Rubisco (LSU) was used as a marker for the soluble phase in an SDS-PAGE gel stained with Coomassie (lower panel). **b** Protease protection assays were performed with *35S*_*pro*_ *:PDI8* microsomes. Samples were either treated (+) or not treated (-) with 50 μg/mL proteinase K (PK) and 0.1 % Triton X-100 (TX), and immunoblot analysis was performed using the anti-PDI8 antiserum. **c** Model of the PDI8 polypeptide oriented in the ER membrane

To further the molecular characterization of PDI8, the orientation of the PDI8 protein in microsomal membranes was investigated. Since overexpression of PDI8 under the CaMV 35S promoter does not lead to mislocalization of PDI8 in stably transformed plants (Additional file 3d), or when transiently expressed in mesophyll protoplasts in the form of the spGFP-PDI8 or PDI8-GFP-KKED fusions (Fig 3b), microsomes were prepared from *35S*_*pro*_*:PDI8* plants due to the strong PDI8 signal these lines exhibited on immunoblots. Separation of the *35S*_*pro*_*:PDI8* protein sample into soluble and microsomal membrane protein fractions revealed that PDI8 was exclusively associated with the microsomal membrane fraction (Fig. 5a, upper panel, lanes 2 and 3 from the left). The microsomes were also tested for the presence of a microsomal marker protein, the soluble ER lumen protein, BiP, by using a polyclonal antibody recognizing BiP. BiP was primarily found in the microsomal fraction (Fig. 5a, middle panel), but a minor amount of BiP was also detected in the soluble protein fraction, which presumably was due to the escape of some proteins from the ER lumen during the mechanical fragmentation of the ER network to produce microsomes. Coomassie staining of an SDS-PAGE gel loaded with equivalent volumes of the total protein, soluble protein, and microsomal protein fractions demonstrated that the large subunit of Rubisco (which serves as a marker for soluble proteins) was present in both the total protein and soluble protein fractions in similar amounts, but was absent in the microsomal fraction (Fig. 5a, lower panel).

Since PDI8 is predicted to contain a single TMD near its C-terminus, we sought to address whether the N-terminal **a-b-b’** region of PDI8 was lumenal (type I membrane protein) or cytoplasmic (type II). *35S*_*pro*_*:PDI8* microsomal membranes were treated with proteinase K to ascertain if the PDI8 N-terminal region was located within the interior of microsomes, and would therefore be protected from degradation. As shown in Fig. 5b, treatment of *35S*_*pro*_*:PDI8* microsomes with protease caused a downward shift in the apparent MW of PDI8 to ~48 kDa (compare lanes 1 and 2), while treatment with both protease and a detergent (Triton X-100) to disrupt the microsomal membranes resulted in the complete degradation of PDI8 (lane 4). Treatment with detergent alone had no effect on the apparent MW of PDI8 (lane 3). Since the C-terminal tail of PDI8 (residues 401–440) contributes a theoretical ~5 kDa to the total MW of PDI8, the observed minor decrease in the MW of PDI8 following proteinase K treatment is consistent with the C-terminal tail being located on the outside of microsomes. A model of the PDI8 polypeptide oriented in the ER membrane is shown in Fig. 5c, indicating that the catalytic domain (a’) and thioredoxin fold domains (b, b’) are oriented into the lumen of the ER.

### Heterologous expression of PDI8 functionally complements the *dsbA*^*−*^ mutation in *E. coli* by reconstituting alkaline phosphatase activity

To gain further insight into the molecular function of PDI8, we examined if PDI8 can functionally complement the *E. coli* oxidative protein folding mutant, *dsbA*^*−*^. The *E. coli* thioredoxin-fold protein, DsbA, plays a crucial role in the oxidative folding of proteins within the bacterial periplasm by catalyzing the formation (dithiol oxidation) of protein disulfide bonds. Loss-of-function mutations of *dsbA* disrupt the proper folding of several proteins, including alkaline phosphatase (PhoA), which in its native state is a homodimer containing two disulfide bonds in each of its subunits [28]. PhoA activity is substantially reduced in a *dsbA*^*−*^ null mutant background, but can be restored by expressing human PDI in the periplasm of *dsbA*^*−*^ cells [14].

**Fig. 6:**
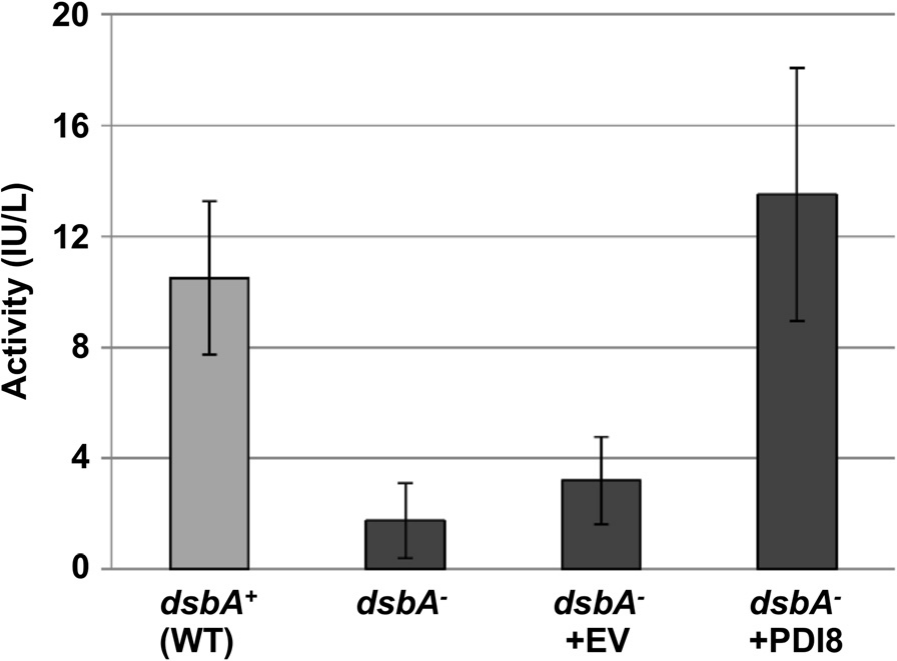
Alkaline phosphatase activity of *E. coli dsbA* ^*−*^ cells expressing the lumenal region of PDI8. PhoA activities were measured from cell lysates obtained from the *dsbA* ^*+*^ strain RI89 (wild-type; WT), the untransformed *dsbA* ^*−*^ strain RI90, and RI90 cells transformed with either the pFLAG-CTS empty vector (+EV) or the pFLAG-PDI8^abb’^ construct (+PDI8). The values are averages of three independent trials, with error bars representing standard deviations

To determine if PDI8 can likewise restore PhoA activity in *dsbA*^*−*^ mutant cells, the coding sequence for the lumenal portion of PDI8 (PDI8^abb’^; Fig. 1b) was cloned into the bacterial expression vector, pFLAG-CTS, between the vector sequences coding for the OmpA signal peptide (for bacterial periplasmic localization) and C-terminal FLAG epitope tag. The resulting plasmid, pFLAG-PDI8^abb’^, was transformed into *E. coli* strain RI90, which harbors the *dsbA* null mutation, *dsbaA1::kan1*. As shown in Fig. 6, PhoA activity in the *dsbA*^*−*^ strain (column 2) or *dsbA*^*−*^ strain transformed with the pFLAG-CTS empty vector (column 3) was substantially reduced relative to the isogenic wild-type (*dsbA*^*+*^) parental control strain RI89 (column 1), whereas *dsbA*^*−*^ cells expressing PDI8^abb’^ exhibited levels of PhoA activity similar to that of wild-type *dsbA*^*+*^ cells (column 4). Thus, the lumenal portion of PDI8 can functionally substitute for the disulfide oxidase role of DsbA in *E coli*.

## Discussion

Because of their conserved structure across eukaryotes, much research attention has focused on classical-type PDIs containing the **a-b-b’-a’** domain organization. In Arabidopsis there are six PDIs with the classical PDI domain arrangement, and each has been shown to localize to the ER lumen [2, 6, 37], although several classical-type PDI isoforms have been shown to also localize to other cellular structures, including protein storage vacuoles, chloroplasts and the nucleus, and to exhibit diverse functions as chaperones and protein foldases [2, 6, 34]. In addition, there are some PDIs that deviate from the **a-b-b’-a’** arrangement, although how these PDIs differ functionally from classical PDIs remains poorly understood.

In this report we describe PDI8, which is the lone member of the novel PDI-B subfamily in Arabidopsis. PDI8 possesses three striking differences that distinguishes it from classical PDIs. First, whereas classical PDIs possess both N-proximal (**a**) and C-proximal (**a’**) thioredoxin catalytic domains, PDI8 only possesses a single, N-proximal **a**-type domain. Second, although PDI8 contains two central redox-inactive **b**-type thioredoxin-fold domains, the **bb’** region of PDI8 does not share sequence homology to the **bb’** region of classical PDIs. Finally, whereas classical PDIs are soluble ER lumen proteins, PDI8 contains a TMD located near its C-terminus. Although the domain arrangement of PDI8 is similar to that of mammalian TMX3, sequence similarity between the two proteins is restricted to their catalytic **a** domains only, implying that they are not orthologous, but instead arose through separate evolutionary events. Indeed, proteins sharing homology to the **bb’** region of PDI8 were only identified in terrestrial plants, and not in representative chlorophyte green algae or non-plant species, indicating that the PDI-B subfamily most likely arose after the evolutionary split between chlorophytes and streptophytes (charophyte algae + terrestrial plants).

Based on the *PDI8*_*pro*_*:GUS* fusion analysis, PDI8 is predicted to play a role in protein folding in young, emerging leaves, in stomata, and in the vasculature of older leaves, roots, and floral organs (Fig. 2). Recently, *PDI8* transcripts were identified in a transcriptomic survey for mobile mRNAs that undergo long-distance transport from shoots to roots [29], and thus the PDI8 protein may be produced in plant tissues beyond those in which the *PDI8* promoter is actively expressed. Indeed, the *PDI8* promoter expression pattern raises the interesting possibility that *PDI8* is expressed in the vasculature specifically for the purpose of mobilizing *PDI8* mRNA to distant tissues via the plant vascular system, possibly to serve as a signal molecule for the coordination of growth processes or for adaptation to environmental stresses in distant plant organs [29]. The PDI8 antiserum developed in this study, combined with proteomic methods, provide an opportunity to investigate this hypothesis and elucidate the cell-specific expression profile in the plant.

To gain further insight into its function, we determined the subcellular location of PDI8. Using two different approaches, we demonstrated that PDI8 localizes to the ER. In immunoelectron microscopy experiments using the PDI8-specific antiserum directed against **b-b’** region, we observed strong labeling of the ER in sections obtained from the shoot apices of wild-type Arabidopsis seedlings (Fig. 4a), with less labeling in sections taken from the root apex (Fig. 4b). This immunolabeling pattern was consistent with the expression pattern of the reporter construct *PDI8*_*pro*_*:GUS*, which exhibited strong expression near the shoot apex (Fig 2a,e), but was not expressed at detectable levels in root tip cells (Fig. 2d). In addition, our analysis of the subcellular distribution patterns of spGFP-PDI8 and PDI8-GFP-KKED in protoplasts indicated that both fusion proteins accumulated in the ER as well (Fig. 3b). Given that PDI8 contains a potential KKxx-type ER retrieval sequence, it is likely that its function is confined to the ER as any PDI8 that would escape the ER membrane would be retrieved by the COPI retrograde pathway. Interestingly, whereas all dicot members of the PDI-B subfamily possessed putative KKxx or xKxx COPI-binding signals at their C-termini, all monocot orthologs instead harbored the C-terminal motif xHxx,. This stands in contrast the other subfamily of integral membrane plant PDIs, PDI-C, in which both monocot and dicot members possess C-terminal xKxx motifs [38]. What effect, if any, the presence of a C-terminal xHxx sequence has on the efficiency of ER retention of monocot members of the PDI-B subfamily in comparison to dicot members remains unclear.

Protease protection experiments indicate that PDI8 is a type I membrane protein with its catalytic **a** domain oriented into the ER lumen (Fig. 5b). Since the members of the PDI-L, PDI-M, and PDI-S subfamilies also localize to the ER in Arabidopsis [37], what specific role does the membrane-bound PDI8 serve in protein folding? There is growing evidence that distinct classes of PDIs, while capable of catalyzing similar reactions *in vitro*, play specialized roles *in vivo* in oxidative protein folding. For example, although the mammalian classical PDI member, PDIA1, can catalyze both disulfide oxidation and disulfide isomerization in peroxiredoxin 4-driven oxidative protein folding, the non-classical PDIA6 (also called P5) and TXNDC5 (thioredoxin domain-containing protein 5; also called ERp46) serve as rapid but promiscuous disulfide oxidases. In contrast, PDIA1 also functions as an isomerase to correct non-native disulfide bonds [25]. This is reminiscent of oxidative protein folding in *E. coli*, where DsbA serves as the principle disulfide oxidase, while DsbC acts as an isomerase [22]. Here we have shown that the **abb’** region of PDI8 can functionally complement the *E. coli dsbA*^*−*^ mutation, indicating that the **a** domain of PDI8 can catalyze the formation (oxidation) of disulfide bonds when heterologously expressed in the bacterial periplasm.

Misfolded proteins can impair cellular processes in a variety of ways, leading to the unfolded protein response (UPR) and ER stress [19, 30]. Due to the important role PDIs serve in catalyzing protein folding, the abnormal accumulation of misfolded proteins within the ER is accompanied by an increase in PDI expression and activity [12]. However, in Arabidopsis only a subset of PDI family members are upregulated by chemical inducers of ER stress [20]. These include half of the PDI-L isoforms (PDI1, PDI5 and PDI6), and all isoforms of PDI-M (PDI9 and PDI10) and PDI-S (PDI11). The absence of PDI8 upregulation in response to ER stress, coupled with its atypical ER membrane localization, suggests that PDI8 functions distinctly from classical PDIs. One possibility is that PDI8 localizes to the ER membrane so that it can rapidly introduce disulfide bonds into newly synthesized secretory proteins as they translocate into the ER lumen. Alternatively, transmembrane PDI8’s role may be to catalyze disulfide bond formation and isomerization specifically in ER transmembrane or membrane-anchored proteins. Substrate proteins with relatively few disulfide bonds have a high probability of being in the proper configuration, whereas proteins with multiple disulfide bonds have a higher probability of containing non-native disulfides, which are subsequently isomerized by a different PDI species. Since the **b’** region serves as the principle binding site for substrates in human PDIA1, the unique **bb’** sequence of PDI8 may allow for the binding of endogenous substrates that are distinct from those of classical eukaryotic PDIs.

## Conclusion

PDI8 is unique to terrestrial plants, is encoded by a single gene in Arabidopsis and is a striking example of a PDI that deviates from the classical **a-b-b’-a’** domain arrangement. Unlike the majority of the PDI family, PDI8 contains a TMD and lacks a second catalytic (**a’**) domain. We demonstrate that PDI8 is a type I endoplasmic reticulum transmembrane protein and a thiol-disulfide oxidase. This work paves the way for studies that will identify the redox-regulated substrates of PDI8 and elucidate its distinct functions in cotyledon guard cells, newly expanding leaves and the vasculature of plants.

## Methods

### Bioinformatic analyses and identification of PDI8 homologs

To identify homologs of PDI8, BLAST (Basic Local Alignment Search Tool) searches were performed against both the National Center for Biotechnology Information (NCBI) non-redundant (nr) protein sequence database (http://blast.ncbi.nlm.nih.gov/Blast.cgi) and the Phytozome v10 (http://phytozome.jgi.doe.gov), using the **bb’** region of PDI8 as the search query sequence due to its absence of homology to the other 13 PDIs from Arabidopsis. Whenever possible, incomplete or incorrectly annotated protein sequences were corrected based on available expressed sequence tag (EST) sequences. All sequences and their corresponding accession numbers are provided in Additional file 4, with alterations to the original source sequences highlighted in yellow.

Signal peptide cleavage site prediction for PDI8 was performed using the program SignalP (v. 4.1) (http://www.cbs.dtu.dk/services/SignalP/; [23]). The predicted locations of TMDs were obtained using the hidden Markov model-based membrane protein topology prediction program, TMHMM (v. 2.0) (http://www.cbs.dtu.dk/services/TMHMM/; [18]). Protein secondary structure predictions for α-helices and β-strands were obtained using the program SPIDER2 (http://sparks-lab.org/yueyang/server/SPIDER2/; [13]).

### Generation of Arabidopsis transgenic plants

A fragment containing the *PDI8* coding sequence was inserted between the cauliflower mosaic virus (CaMV) 35S promoter and nopaline synthase (*NOS*) terminator sequences of binary vector *pCAMBIA1302* to create the *PDI8* over-expression construct, *pC1302[35s*_*pro*_*:PDI8]*. The *PDI8* coding sequence was amplified from first-strand cDNA prepared from 7-day-old Arabidopsis ecotype Columbia-0 (Col-0) seedlings using a forward primer containing an engineered SphI restriction site (5’-TCG GCA TGC GTT CGT TAA AGT TAC TCC TTT G-3’), and a reverse primer containing a BstEII site (5’-AAG GGT CAC CAA ACT AGT CCT CTT TTT TGT CAC-3’). The incorporated restriction sites in these primer sequences (and all subsequent primers described in this report) are underlined. The *PDI8* cDNA fragment was ligated between the NcoI and BstEII restriction sites of *pCAMBIA1302*. For antisense expression of *PDI8*, a PDI8 cDNA fragment was amplified from cDNA (as above) using a forward primer containing a BstEII restriction site (5’-AAA TGG TGA CCT CAT GAG ATC GTT AAA GTT ACT CCT TTG TTG-3’), and a reverse primer with an SpeI site (5’-AAG TGG GTC AAC ACT AGT CCT CTT TTT TGT CAC T-3’). The cDNA fragment was ligated in the antisense orientation between the SpeI and BstEII cloning sites of *pCAMBIA1302* to generate the construct *pC1302[35S*_*pro*_*:antisensePDI8*].

Promoter expression studies were performed using stably transformed transgenic lines harboring the construct *PDI8*_*pro*_*:GUS*, which contains a 2.3-kb *PDI8* promoter fragment transcriptionally fused to the β-glucuronidase (GUS) reporter gene, *gusA*. The *PDI8* promoter fragment was amplified from Arabidopsis (Col-0) genomic DNA, using a forward primer with a SacI site (5’- TTT GAG CTC GTA GAA GTT TGC TTG AAT ATT CA-3’) and a reverse primer with an NcoI site (5’-AAC CCA TGG CGA TCT GAT TTT CAG ACC AAA C-3’). The *gusA* gene was amplified from *pCAMBIA1304* using a forward primer with an NcoI site (5’-TGA CCA TGG TAG ATC TGA CTA GTT TAC GTC-3’) and a reverse primer with a BstEII site (5′-CTC CGG TCA CCT ATT GTT TGC CTC CCT GCT GCG-3′). The *PDI8*_*pro*_*:GUS* fusion was assembled in *pCAMBIA1302* by cloning the *gusA* PCR fragment between the NcoI and BstEII sites of the vector to create the intermediate construct *pC1302[35S*_*pro*_*:GUS]*. The *PDI8* promoter fragment was then cloned between the SacI and NcoI sites of *pC1302[35s*_*pro*_*:GUS]* to produce the final construct, *pC1302[PDI8*_*pro*_*:GUS]*.

The *pC1302[35S*_*pro*_*:PDI8]* and *pC1302[PDI8*_*pro*_*:GUS]* constructs were introduced into wild-type Arabidopsis (Col-0) plants by Agrobacterium-mediated transformation, using the floral dip method [7]. Initial transformants were obtained by selecting for hygromycin resistance in the T_1_ generation, and the presence of an intact transgene determined by PCR. Homozygous transgenic lines were subsequently identified by screening for the occurrence of 100 % hygromycin-resistance in the T_3_ generation.

### GUS expression analysis

*PDI8*_*pro*_*:GUS* seedlings were grown vertically on 1/2× LS agar plates [0.8 % (w/v) Gellan Gum (Sigma-Aldrich, St. Louis, MO), 1/2x Linsmaier & Skoog media (Caisson Laboratories, Smithfield, UT) and 1.5 % (w/v) sucrose] for 7 or 14 days at 22 °C under a 16 h-light/8 h-dark cycle. Shoot inflorescences were obtained from 6-week-old *PDI8*_*pro*_*:GUS* plants grown on Farfard Super-Fine Germinating Mix (Sun Gro Horticulture, Agawam, MA) under a 16 h-light/8 h-dark cycle at 25 °C. GUS staining was performed as described [17]. Briefly, the tissue samples were fixed in 90 % ice-cold acetone for 20 min at 25 °C, then washed with staining buffer (50 mM sodium phosphate buffer (pH 7.0), 0.2 % Triton X-100, 2 mM potassium ferrocyanide, and 2 mM potassium ferricyanide) three times on ice, then submerged in staining buffer containing 1 mM 5-bromo-4-chloro-3-indoxyl-β-D-glucuronide cyclohexylammonium salt (X-gluc). The tissues were vacuum infiltrated briefly, then incubated O/N at 37 °C. After staining, the samples were incubated in 70 % ethanol to extract soluble pigments, repeating with fresh 70 % ethanol as necessary. Images of GUS staining in roots and stomata were acquired on an Olympus BX-51 upright microscope, with the samples mounted on glass slides in 50 % glycerol. All other images were taken on an Olympus SZX-12 stereomicroscope, with samples submerged in 70 % ethanol in a petri dish.

### Transient expression of spGFP-PDI8 in protoplasts

The creation of the ER marker construct *pBL(35S*_*pro*_*:ER-mCherry)*, and the unfused green fluorescent protein (GFP) control construct *pBL(35S*_*pro*_*:GFP(S65T))*, was described previously [6]. The construct *pBL(35S*_*pro*_*:spGFP-PDI8)* was generated by cloning the following arrangement of DNA sequences between the KpnI and BstEII sites of *pBL(35S*_*pro*_*:GFP(S65T))*: a *CaMV 35S* promoter fragment (KpnI/XhoI), a PDI8 signal peptide coding sequence-*GFP(S65T)* fragment (XhoI/XmaI), and a PDI8 mature protein cDNA fragment (XmaI/BstEII). The *CaMV 35S* promoter fragment was amplified from *pCAMBIA1302* using a forward primer with a KpnI site (5’-TTC AGG GTA CCT TCA TGG AGT CAA AGA TTC A-3’), and a reverse primer with an XhoI site (5’-ATC TAC TCG AGT CAA GAG TCC CCC GTG-3’). The *GFP(S65T)* fragment, modified to include the signal peptide sequence at the N-terminus of GFP, was amplified from plasmid *HBT95::sGFP(S65T)-NOS* using a forward primer with an XhoI site (5’-TTT CTC GAG ATG CGT TCG TTA AAG TTA CTC CTT TGT TGG ATC TCG TTT CTT ACG TTA TCA ATC TCA ATC TCT GCA TCG TCA ATG GTG AGC AAG GGC GAG GAG CTG-3’), and a reverse primer with an XmaI site (5’-AAA CCC GGG CTT GTA CAG CTC GTC CAT GC-3’). The PDI8 mature protein cDNA fragment was amplified from a full-length *PDI8* cDNA clone using a forward primer with an XmaI site (5’-ATA CCC GGG TCG TCA GAT GAT CAA TTC ACC CTC-3’) and a reverse primer with a BstEII site (5’-AAG GGT CAC CAA ACT AGT CCT CTT TTT TGT CAC TAG-3’). The construct *pBL(35S*_*pro*_*:PDI8-GFP-KKED)* was generated by replacing the *spGFP-PDI8* coding sequence of *pBL(35S*_*pro*_*:spGFP-PDI8)*, between restriction sites XhoI and BstEII, with a full-length *PDI8* cDNA fragment (XhoI/XmaI) and a *GFP(S65T)-KKED* fragment (XmaI/BstEII). The *PDI8* cDNA fragment was amplified from a *PDI8* cDNA clone using a forward primer with an XhoI site (5’-CAG CTC GAG ATG CGT TCG TTA AAG TTA C-3’) and a reverse primer with an XmaI site (5’-ACA CCC GGG GTC CTC TTT TTT GTC ACT AGG CT-3’). The *GFP(S65T)* fragment, modified to include the KKED putative retention signal of PDI8, was amplified from plasmid *HBT95::sGFP(S65T)-NOS* using a forward primer with an XmaI site (5’-TAG TCC CGG GAT GGT GAG CAA GGG CGA GGA-3’), and a reverse primer with a BstEII site (5’-AGG ATG GTC ACC TAA TCC TCT TTT TTG CCG TGA GTG ATC-3’).

The procedure for isolating and transfecting protoplasts was adapted from Yoo et al. [36] and Wu et al. [35]. The abaxial epidermis of rosette leaves from four-week-old *Arabidopsis* plants was removed using the tape-sandwich method [35]. Mesophyll cells were released by incubating the peeled leaves in 10 mL of enzyme solution (1.5 % cellulase R10, 0.4 % macerozyme R10, 0.4 M mannitol, 20 mM KCl, 20 mM MES, pH 5.7) for 3 h, then mixed gently with 10 mL of W5 solution (154 nM NaCl, 125 mM CaCl2, 5 mM KCl, 2 mM MES, pH 5.7). The protoplasts were gently centrifuged at 100 *g* for 2 min, resuspended in fresh W5 solution to a density of 2 × 10^5^/mL, and incubated on ice for at least 30 min. The W5 solution was then removed, and the protoplasts resuspended in MMg solution (0.4 M mannitol, 15 mM MgCl_2_, 4 mM MES, pH 5.7) to a density of 2 × 10^5^/mL. The protoplasts were transfected by gently mixing 200 μL of protoplasts in MMg solution with 20 μL of plasmid DNA solution (containing ~20 μg of each construct in H_2_O), and 220 μL of PEG solution (40 % PEG, 0.2 M mannitol, 100 mM CaCl_2_). After incubating at 25 °C for 5-10 min, transfection was stopped by adding 0.8 mL W5 solution. The protoplasts were centrifuged at 100 *g* for 2 min, and then resuspended in 1 mL WI solution (0.5 M mannitol, 20 mM KCl, 4 mM MES, pH 5.7). The transfected protoplasts were incubated in the dark at 22 °C for 18 h to allow for transgene expression. Fluorescence was visualized using an Olympus FV-1000 laser scanning confocal microscope at the Biological Electron Microscope Facility (University of Hawaii at Manoa, Honolulu, HI). The excitation/emission filters utilized for fluorescence detection were 488/505–525 nm for GFP(S65T) and 543/585–615 nm for mCherry.

### Anti-PDI8 antibody production

Affinity-purified polyclonal rabbit antibodies recognizing PDI8 were generated commercially through YenZym Antibodies, LLC (San Francisco, CA), using a truncated form of recombinant PDI8 as the antigen for both rabbit immunization and affinity purification of the antiserum. For production of the truncated PDI8 protein, a cDNA fragment encoding the central **b-b’** region of PDI8 (PDI8^bb’^, corresponding to residues 138-377 of the PDI8 preprotein sequence) was amplified by RT-PCR using a forward primer with an NdeI site (5’-GCC TAC GCA TAT GGT TGC TCC AGA TGT GCG G-3’) and reverse primer with a BamHI site (5’-CGT GGA TCC CTA TGA GTT GAT AAA TCC CAT GAA-3’). The *PDI8*^*bb’*^ cDNA fragment was ligated between the NdeI and BamHI sites of the bacterial expression vector pET-15b (EMD Millipore, Billerica, MA), placing the *PDI8*^*bb’*^ sequence in-frame with the 6xHis-tag of pET-15b. Expression of PDI8^bb’^ was induced in *Escherichia coli* strain BL21(DE3) for 5 h at 28 °C by the addition of 0.2 mM IPTG. After induction, the *E. coli* cells were harvested by centrifugation and lysed using BugBuster Protein Extraction Reagent (EMD Millipore). The His-tagged PDI8^bb’^ protein was purified from the bacterial lysate by nickel affinity chromatography.

### Transmission electron microscopy and immunolabeling

For immunogold labeling analysis, developing roots and apical buds were preserved by high-pressure freezing/freeze-substitution techniques as described in [6]. For immunolabeling, 80 nm thick sections from Lowicryl HM20 resin embedded specimens were placed on formvar-coated gold or nickel slot grids and blocked for 30 min with 2 % (w/v) non-fat dried milk solution in 0.01 M phosphate-buffered saline pH 7.2 containing 0.1 % Tween-20 (PBST). The sections were washed and then incubated with a 10-fold dilution of the primary antibody, anti-PD8, for 2 h at RT. Sections were washed and transferred to a 25-fold dilution of secondary antibody goat anti-rabbit IgG-conjugated to 10 or 15 nm gold particles (Ted Pella, Inc) for 2 h at RT. Sections were washed and then stained with uranyl acetate solution for 2 min and lead citrate for 4 min. All observations were performed using a Hitachi H-7000 transmission electron microscope operated at 80 KV (Hitachi USA, OH).

### Preparation of microsomal membranes and protease protection analysis

For extraction of microsomal membranes, *35S*_*pro*_*:PDI8* Arabidopsis seedlings were grown vertically on 1/2× LS agar plates under a 16 h-light/8 h-dark cycle at 22 °C. 7-day-old seedlings were homogenized with a chilled mortar and pestle in ice-cold extraction buffer [40 mM HEPES pH 7.5, 0.4 % polyvinyl polypyrrolidone (PVP), 1 mM MgCl_2_, 10 mM KCL, and 0.4 M sucrose], at a ratio of 1.5 μL extraction buffer per 1 mg of plant tissue. To remove insoluble debris, the homogenate was centrifuged twice at 1000 *g* and 4 °C for 3 min, collecting the supernatant after each spin. The total protein homogenate was separated into microsomal and soluble protein fractions by centrifuging as 150 μL aliquots at 21,000 *g* and 4 °C for 1.5 h [1]. The microsomal pellets were washed once with 150 μL of fresh extraction buffer, recovering the microsomes by spinning at 21,000 *g* and 4 °C for 45 min and removing the supernatant. Finally, the microsomal pellets were resuspended in a volume of fresh extraction buffer equivalent to the original sample volume (i.e. 150 μL).

For immunoblot detection of PDI8 and BiP, protein samples were separated by SDS-PAGE (10 % polyacrylamide gels) and transferred onto nitrocellulose membranes. An equivalent amount (by volume) of the *35S*_*pro*_*:PDI8* total and fractioned protein samples were loaded, equaling ~20 μg protein in the unseparated homogenate, ~14 μg protein in the soluble fraction, and ~7 μg protein in the microsomal fraction. Immunoblot analysis of PDI8 was performed using the anti-PDI8 antiserum at 1:100 dilution, and an anti-rabbit horseradish peroxidase (HRP)-conjugated secondary antibody at 1:2000 dilution supplied in the Amersham ECL Western Blotting Detection Kit (GE Healthcare Bio-Sciences, Pittsburgh, PA). Detection of BiP was performed using the goat anti-BiP primary antibody aC-19 (Santa Cruz Biotechnology, Inc., Dallas, Tx) at 1:1000 dilution, and a donkey anti-goat HRP-conjugated secondary antibody (Santa Cruz Biotechnology, Inc.) at 1:3000 dilution.

To determine the membrane topology of PDI8, *35S*_*pro*_*:PDI8* resuspended microsomes were incubated at 37 °C for 30 min in extraction buffer alone (negative control), or with 50 μg/mL proteinase K and/or 0.1 % Triton X-100. Each reaction contained ~0.36 μg/μL microsomal protein in a total volume of 60 μL. Proteinase K digestion was stopped by adding 5 mM PMSF to all samples. SDS-PAGE and immunoblot detection of PDI8 was performed as described above, with each lane loaded with 20 μL of sample (~7.2 μg of microsomal protein).

### Alkaline phosphatase activity assay

A construct for the heterologous expression of PDI8 in *E. coli* was generated by cloning the coding sequence for the lumenal portion of PDI8, containing the catalytic **a** domain and redox-inactive **b** and **b’** domains (PDI8^abb’^, Fig. 1) between the XmaI and SalI restriction sites of the bacterial expression vector pFLAG-CTS (Sigma-Aldrich, St. Louis, MO). The *PDI8*^*abb’*^ gene fragment was amplified from a full-length *PDI8* cDNA using a forward primer with an XmaI site (5’-TGT CCC GGG AGA TGA TCA ATT CAC CCT CGA C-3’) and a reverse primer with a SalI site (5’-AAT GTC GAC CAT TGA GTT GAT AAA TCC CAT G-3’).

The *E. coli* strains RI89 (*dsbA*^*+*^) and RI90 (*dsbA::kan1*; RI89 genetic background) were obtained from the *E. coli* Genetic Stock Center (Yale University, New Haven, CT). The pFLAG-PDI8^abb’^ construct and pFLAG-CTS empty vector were transformed into strain RI90. To measure alkaline phosphatase (PhoA) activity, the cells were grown at 37 °C in M9 minimal media to an OD_600_ of 0.4–0.6, harvested by centrifugation, washed once with 50 mM Tris-HCl (pH 8.0), and lysed with 0.2 % Triton X-100. PhoA activity was determined using the QuantiChrom Alkaline Phosphatase Assay Kit (BioAssay Systems, Hayward, CA). Briefly, 150 μL of working solution (5 mM magnesium acetate, and 10 mM *p*-nitrophenyl phosphate in supplied assay buffer, pH 10.5) was added to 50 μL of lysed cells. After quickly mixing, the initial OD_405_ (t = 0) was measured for each sample, and then re-measured after 4 min (t = 4). PhoA activity (IU/L) was calculated from the OD_405_ values as described in the kit. The activities reported are averages (±standard deviation) derived from three independent trials.

## Additional files

Additional file 1: Online Resource 2. Relative levels of PDI8 transcripts across various plant organs of Arabidopsis. An electronic fluorescent pictograph depicting the relative expression level of *PDI8* across different Arabidopsis tissues based on publicly available microarray data. (JPG 587 kb) Additional file 2: Online Resource 3. Validation of anti-PDI8 antiserum. a Immunoblot detection of recombinant PDI8^bb’^ by anti-PDI8 antiserum. b Immunoblot detection of PDI8 in WT and *35S*_*pro*_ *:PDI8* overexpression lines by the anti-PDI8 antiserum. c Immunodetection of PDI8 expressed in the *E. coli* strain RI89 (*dsbA* ^*+*^ mutant) from the pFLAG-PDI8^abb’^ construct. (TIF 3362 kb) Additional file 3: Online Resource 4. Validation of antibody specificity in tissues for microscopy. a Section of a root apical cell from the *pdi8* antisense line, AS1, stained with anti-PDI8 antiserum. b Section of a shoot apical cell from AS1, stained with anti-PDI8 antiserum. c Section of a WT root apical cell, labeled with rabbit pre-immune serum. d Section of a root apical cell from the line OE1 labeled with anti-PDI8 antiserum. (JPG 9403 kb) Additional file 4: Online Resource 1. Sequences of PDI-B subfamily proteins identified by database searches. A compilation of the deduced products of *PDI-B* genes found in available sequenced plant genomes. (DOCX 134 kb)

## Abbreviations

BiP: Binding immunoglobulin protein
BLAST: Basic local alignment search tool
CaMV: Cauliflower mosaic virus
COPI: Coat protein I
Dsb: Disulfide bond formation protein
eFP: Electronic fluorescent pictograph
ER: Endoplasmic reticulum
Erv: ER vesicle protein
EST: Expressed sequence tag
GFP: Green fluorescent protein
GUS: β-glucuronidase
HRP: Horseradish peroxidase
MEE8: Maternal effect embryo arrest 8
MW: Molecular weight
NCBI: National center for biotechnology information
NOS: Nopaline synthase
OmpA: Outer membrane protein A
PDI: Protein disulfide isomerase
PEG: Polyethylene glycol
PhoA: Alkaline phosphatase
PMSF: Phenylmethylsulfonyl fluoride
RT-qPCR: Quantitative reverse transcription polymerase chain reaction
Rubisco: Ribulose-1,5-bisphosphate carboxylase/oxygenase
SDS-PAGE: Sodium dodecyl sulfate polyacrylamide gel electrophoresis
TAIR: The arabidopsis information resource
TMD: Transmembrane domain
TMX3: Thioredoxin-related membrane protein 3
TXNDC5: Thioredoxin domain-containing protein 5
UPR: Unfolded protein response
WT: Wild type
X-gluc: 5-bromo-4-chloro-3-indoxyl-β-D-glucuronide cyclohexylammonium salt.
