# Supplementary figures and images for: Arabidopsis protein disulfide isomerase-8 is a type I endoplasmic reticulum transmembrane protein with thiol-disulfide oxidase activity
Source: BMC Plant Biol. 2016 Aug 22;16(1):181. doi: 10.1186/s12870-016-0869-2 (PMC4994283; doi:10.1186/s12870-016-0869-2)

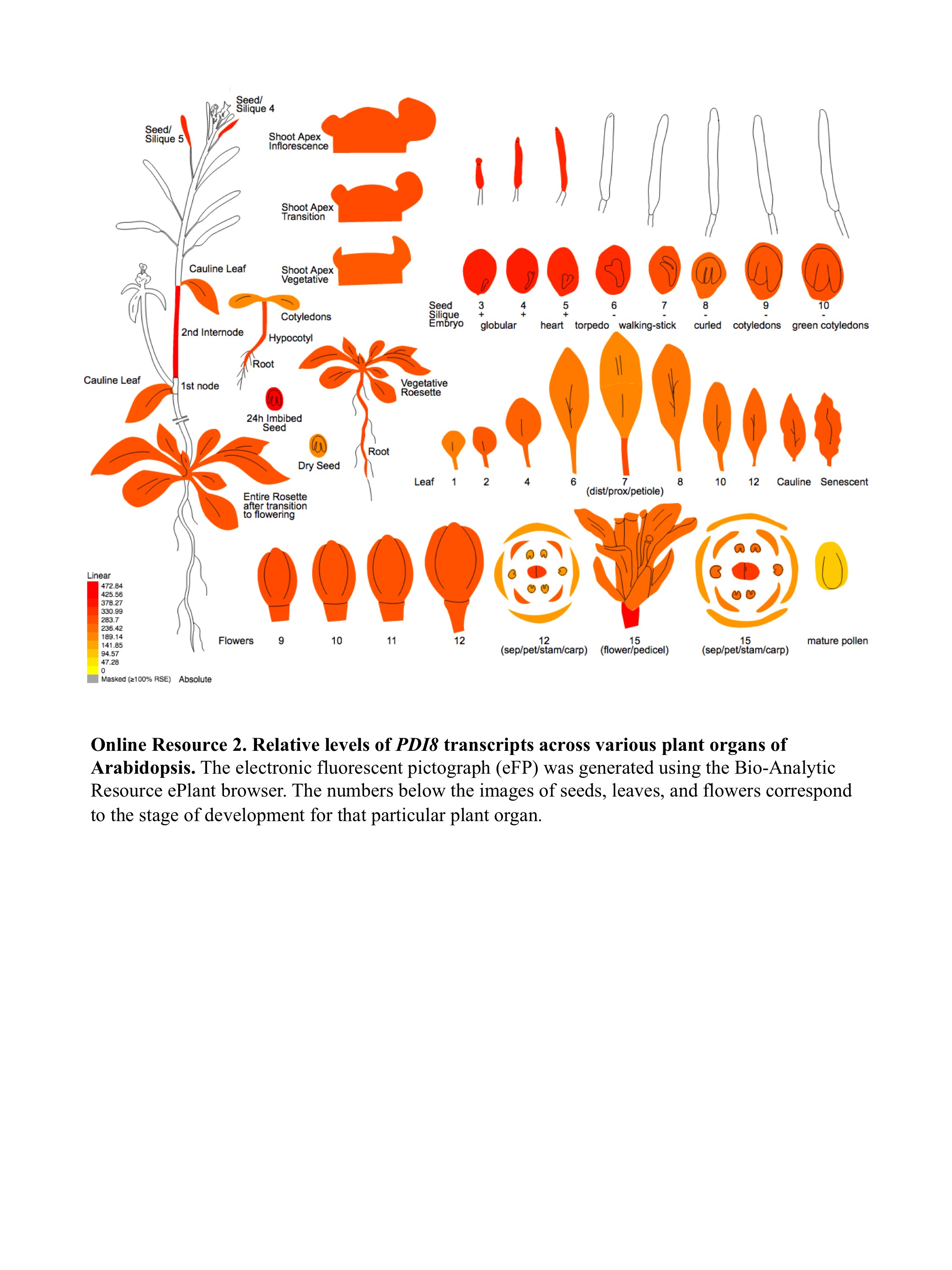

Supplement: Additional file 1: Online Resource 2. — Relative levels of PDI8 transcripts across various plant organs of Arabidopsis. An electronic fluorescent pictograph depicting the relative expression level of PDI8 across different Arabidopsis tissues based on publicly available microarray data. (JPG 587 kb) [file 12870_2016_869_MOESM1_ESM.jpg]

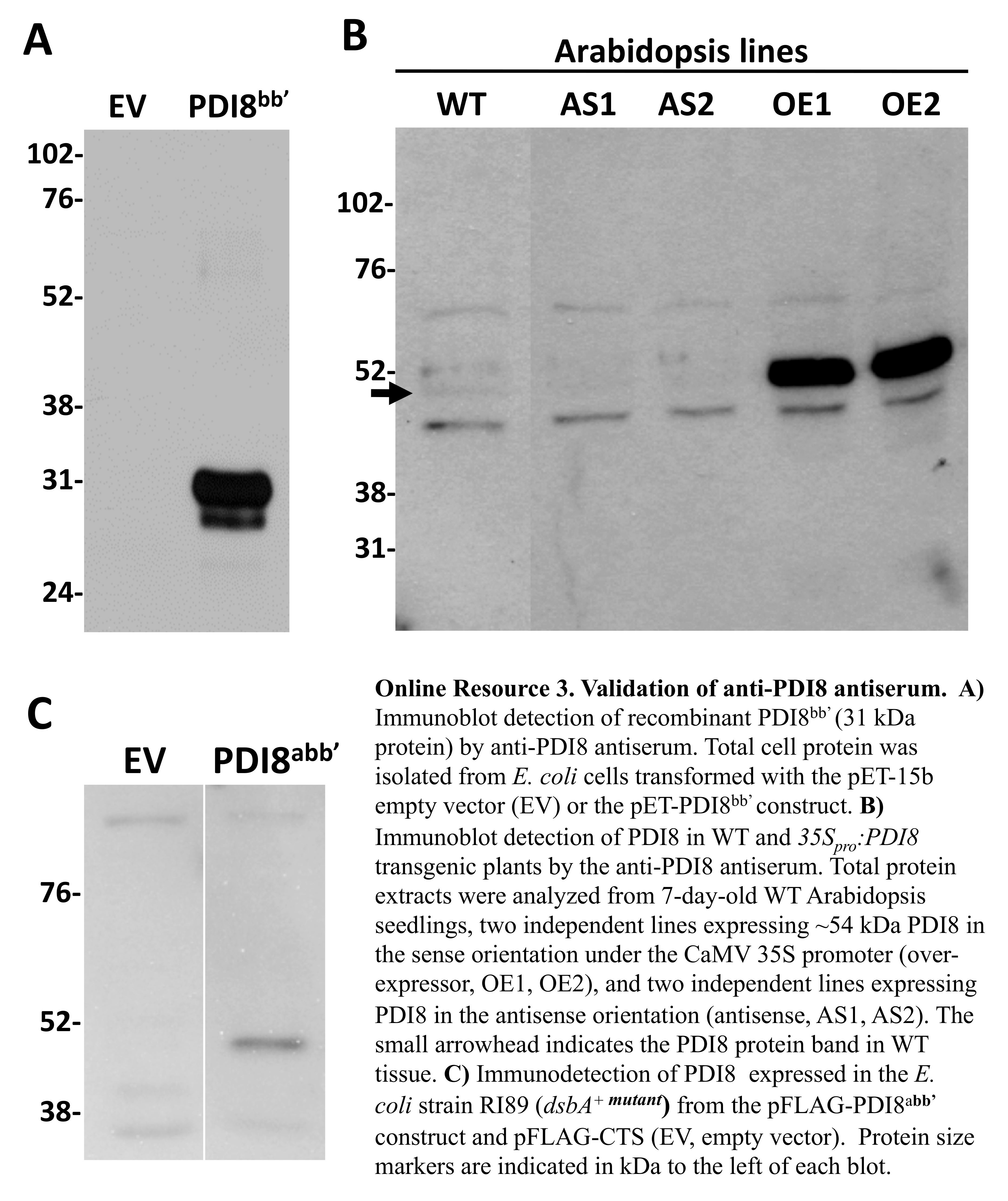

Supplement: Additional file 2: Online Resource 3. — Validation of anti-PDI8 antiserum. a Immunoblot detection of recombinant PDI8bb’ by anti-PDI8 antiserum. b Immunoblot detection of PDI8 in WT and 35S pro :PDI8 overexpression lines by the anti-PDI8 antiserum. c Immunodetection of PDI8 expressed in the E. coli strain RI89 (dsbA + mutant) from the pFLAG-PDI8abb’ construct. (TIF 3362 kb) [file 12870_2016_869_MOESM2_ESM.tif]

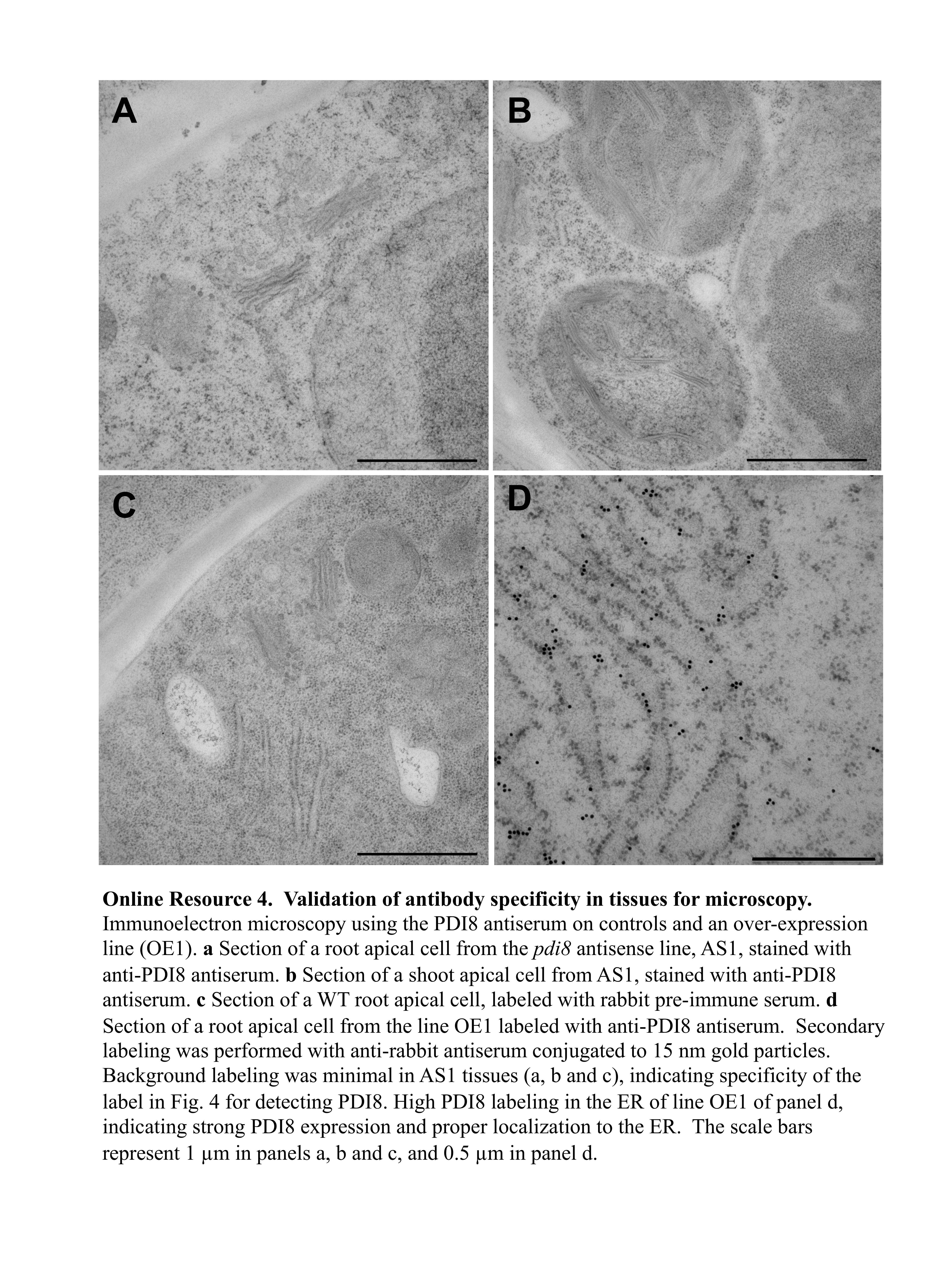

Supplement: Additional file 3: Online Resource 4. — Validation of antibody specificity in tissues for microscopy. a Section of a root apical cell from the pdi8 antisense line, AS1, stained with anti-PDI8 antiserum. b Section of a shoot apical cell from AS1, stained with anti-PDI8 antiserum. c Section of a WT root apical cell, labeled with rabbit pre-immune serum. d Section of a root apical cell from the line OE1 labeled with anti-PDI8 antiserum. (JPG 9403 kb) [file 12870_2016_869_MOESM3_ESM.jpg]
