# Supplementary material for: Arabidopsis protein disulfide isomerase-8 is a type I endoplasmic reticulum transmembrane protein with thiol-disulfide oxidase activity
Source: BMC Plant Biol. 2016 Aug 22;16(1):181. doi: 10.1186/s12870-016-0869-2 (PMC4994283; doi:10.1186/s12870-016-0869-2)
Supplement: Additional file 4: Online Resource 1. — Sequences of PDI-B subfamily proteins identified by database searches. A compilation of the deduced products of PDI-B genes found in available sequenced plant genomes. (DOCX 134 kb) [file 12870_2016_869_MOESM4_ESM.docx]

**Online Resource 1.** Sequences of PDI-B subfamily proteins identified by database searches. Corrections made to the original sequences due to available EST data or an alternate gene prediction model are highlighted in yellow.

**PDI-B Subfamily Proteins**

**NCBI Reference Sequence: NP_564462.1**

>A.thalianaPDI8

MRSLKLLLCWISFLTLSISISASSDDQFTLDGTVLELTDSNFDSAISTFDCIFVDFYAPWCGHCKRLNPE

LDAAAPILAKLKQPIVIAKLNADKYSRLARKIEIDAFPTLMLYNHGVPMEYYGPRKADLLVRYLKKFVAP

DVAVLESDSTVKEFVEDAGTFFPVFIGFGLNESIISGLGRKYKKKAWFAVSKEVSEDTMVSYDFDKAPAL

VANHPTYNEHSVFYGPFEDGFLEEFVKQSFLPLILPINHDTLKLLKDDERKIVLTIVEDETHESLEKLYK

ALRAAAHANRDLVFGYVGVKQFEEFVDSFHVDKKTNLPKIVVWDGDEEYDQVTGIETITQEEDHLTQVSR

FLEGYREGRTEKKKINGPSFMGFINSMIGIRSVYILVFLVAVIMMLRSLGQVEEPTGVRTATAVRERVDQ

ATTVPEDESSEHKPSDKKED

**NCBI Reference Sequence: XP_003579772.1**

>B.distachyonPDI-B1

MATTLPPLPLLCLLPLLLVAFATAGGGSGGGGGENFPRDGRVIDLDDSNFEAALSSIDFLFVDFYAPWCG

HCKRLAPELDEAAPVLAGLSEPIMVAKVNADKYRKLGSKYGVDGFPTLMLFIHGVPIEYTGSRKADLLVR

NLKKFVAPDVSILESDSAIKSFVENAGTSFPMFIGFGVNESLITEYGGKYKKRAWFAIAQDFSEELMMAY

GFDKAPALVALHPKYNEQSVFYGPFEGRFLEDFIRQSLLPLTVPINTETLKLLDDDDRKVVLAILEDDSD

ENSAQLVTVLRSAANANRDLVFGYVGVKQWEEFVETFDVSKSSQLPKLLVWDRNEEYEQVDGSERLEEGD

QASQISQFLEGYRAGRTTKKKVSGPSFMGFMHSLVSMNSLYILMFVVALLGVMLYFSGQDDTPQLRRIHD

E

**NCBI Reference Sequence: XP_003572607.1**

>B.distachyonPDI-B2

MAAGKPLSLRRLLPLLALVLVLVLLPTTCVSSGGGEPAGFQIPQDGSVVELDDSNFEAAAAAVDFLFVDF

HAPWCGHCKRLSPQLDEAAPVLAGLSTPVVVAKVDAEKYKKLGSKYGVDGFPTLMLFDHGVPTEYTGSRK

ADLLIQSLKKLVAPDFSVLGSDSAIKSFVQDAGVGFPLFIGFGVDESSIVEYGTRYKRKAWFAAAKDFSE

DMMVVYDFDKIPALVSLNPKYNEQSVFYGPFEGTFLEDFIRQSLLPLTVPINAETVKMLKDDERKVVLTV

LEDESDENSMQLIKVLRSAANANHDLVFGYVGVKQWEEFTEPFHDSESSRLPRMVVWDRNEEYEVVQGSE

NLEDGDHGSQISRFLEGYRAGRTTKKKLGGRSPTILGVNAMYILLFLVAVLVVLMYFSGQGEEDRWPARA

HQE

**NCBI Reference Sequence: XP_006307553.1**

>C.rubellaPDI-B

MRSLKLLLCWISFLTLSISLSASSDDEFTIDGTVLELTDSNFDSAISSFDCIFVDFYAPWCGHCKRLNPE

LDAAAPILAKLKKPIVIAKLNADKYSRLARKHEIDAFPTLMLYNHGVPMEYYGPRKADLLVRYLKKFVAP

DVAVLESDSAVKEFVEDAGTFFPVFIGFGLNESLISGLGGKYKKKAWFAVAKDASEDVMVSYDFDKAPAL

VAKHPTYNEHSVFYGPFEDGFLEEFVKQSFLPLILPINQDTLKMLKDDERKIVLTIVEDETHESLGKLTK

ALRAAAHANRDLVFGYVGVEQFEEFADSFHADKKTNLPKIVVWDGDEEYDQVTGIETISQEEDHLTQVSR

FLEGYREGRTERKKIKGPSLMGFINSMIGIRSVYVIVFFVAIIMMLRSLGQVDEPDRVRTTTAERVDQTT

SVPEGETSEHMASDKKED

**NCBI Reference Sequence: XP_006403585.1**

>E.salsugineumPDI-B

MRSLALVFCWTSFSALSVSLSASSSDDQFNVNGKVLELTDSNFDSAISTFDCIFVDFYAPWCGHCKRLNP

ELDAAAPVLAKLKKPIVIAKLNADKYSRLARKLEIDAFPTLMLYNHGVPMEYYGPRKADLLVRYLKKFVA

SDVAVLESDAAVKDFVEDAGTFFPVFIGFGLNESLITALGAKYKKKAWFAVAKDISEDVMVSYDFDKAPA

LIAKHPSYNERSVFYGPFEDGFLEDFVKQNFLPLVLPINQDTLKLLKDDERKMVLTIVEDETHESLGKLI

KALRAAAYANRDLVFGYVGVEQFEEFADSFHADKKAKLPKIVVWDGDEEYDEVNGIETISHEEDHLTQVS

RFLEGYREGRTEKKRIKGPSLMGFINSMIGIRSVYIIVFLVAVIMMLRSLGQVEEPARARTSEAACERVD

QTTSVSQGETSEHKPRDKED

**NCBI Reference Sequence: XP_003536390.1**

>G.maxPDI-B

MRMKRVFVVYSVLLLLVQFWLGEAETFSVDGKVLVLDESNFDSAIASFDHILVDFYAPWCGHCKRLSPEL

DAAAPVLATLKEPIIIAKVDADKHTRLAKKYDVDAYPTILLFNHGVPTEYRGPRKADLLVRYLKKFSASD

VSILDSDSAVNMFVEEAGTFFPIYIGFGLNSSVLEKFGIKYKKNAWFSVAKDFSEDLMVLHDFDKIPALV

SLNPQYNERNTFYGPFEEDFLEDFVRQNLIPLAVPVSYETLKLMKADGRKIVLTIVEDEDEETTRELIKL

LKAAASANRDLIFGYVGVKQMEEFAENFDIDTKLPKMVIWDKSDDYLSVVDSETIEGEDQGTQITKFLEG

YREGRTIKKTFSGPSLMRFIHRSFDIRMVYIIVFVVAVLMLIQTFSKGGDEYQSVPNQVQTDHAISSVSE

AENNEYKPGDKED

**NCBI Reference Sequence: XP_013469538.1**

>M.truncatulaPDI-B

MRICFVIFCIVSLIHFCRAEPLSVDGKVLILDESNFDSAISSFDHILVDFYAPWCGHCKRLSPELDAAAP

VLAALKEPILIAKVDADKHTSLARKHDVDAYPTILLFNHGVPTEYRGPRKADLLVRYLKKFAASDVSILD

SDSAVNNFVEEAGTFFPVYIGFGLESSMIEKFGKKYKKNAWFSVAKDFSEDLMVTYDFDKIPALVSLNPL

YNERNTFYGPFEDDFLEDFIKQNLIPLAVPVSYETLKLMKADGRKIVLTIVDDESEESSKELVKLLRAAA

SANRDLIFGYVGVKQLDEFADKFDTTTKLPKMVIWDKEDEYLSVVGSESIEAEDQGTQITKFLEGYREGR

TVKKSFSGPSLMQFIHRSFDIRMVYIVVFMIAVLMLIQTLGSKGDDGEYQRVPNQDKVNQPSSSTSEGES

KEYKEGDKED

**NCBI Reference Sequence: NP_001052831.2**

>O.sativaPDI-B1

MAATTTRPLPLLLLLLLPPLLLLLLSFHAAAAAAAEEFPRDGRVIELDESSFEAALGAIDYLFVDFYAPW

CGHCKRLAPELDEAAPVLAGLSEPIIVAKVNADKYRKLGSKYGVDGFPTLMLFIHGVPIEYTGSRKADLL

VRNLNKFVAPDVSILESDSAIKSFVENAGTSFPMFIGFGVNESLIAGYGGKYKKRAWFAVAKDFSEDFMV

TYDFDKVPALVSLHPKYKEQSVFYGPFEGSFLEDFIRQSLLPLTVPINTETLKMLDDDDRKVVLAILEDD

SDETSSQLVKVLRSAANANRDLVFGYVGIKQWDEFVETFDISKSSQLPKLIVWDRNEEYEVVEGSEKLEE

GDQASQISQFLEGYRAGRTTKKKVSGPSFMGFLNSLVSLNSLYILICVFALLGVMIYFTGQDDTPQVRRA

HEE

**NCBI Reference Sequence: NP_001047093.1**

>O.sativaPDI-B2

MGKPTLPPVVVVVVLLLLVVVLPATTCGADAGGGGEAEEFQIPRDGRVLELDDGNFDAAVRAAGLLFVDF

YAPWCGHCKRLAPQLDEAAPVLAGLSTPIVVAKVNADKYKKLGSKYGVDGFPTLMLFDHGTPTEYTGSRK

ADLLVENLKKLVAPDVSVLESDSAIKSFVEDAGMGFPLFLGFGVDESLIVEYGAKYKNRAWFSVAKDFSE

DMMVFYDFDKVPALVSVNPKYREQSIFYGPFDDGAFLEDFIRNSLLPLVVPMNRETVKMLNDDGRKVVLM

ILQDDESDENSPRLIKVLRSAASANRDLVFGYVGVNQWEEFTETFDVKSSELPTMIVWDKKEEYEIVEGS

ERLEEGDYGSQISRFLEGYRAGRTIKKKVGDRSPTLLGVNAVYILVFLVAVLVLLMYFSGQGEEDQRPRQ

RAHED

**NCBI Reference Sequence: XP_001762511.1**

>P.patensPDI-B1

MAIVVKVLALVLLLRSAVSLAAVQLDDLEESPDLGKVVELTDASIEAALNRHEYILIDFYAPWCKHCQSL

SPQLDQAAPFLADGEPSIVVAKLNADKYRTMAEKYDISFYPTLKFFANGYPTDYDGPHSANALVSHVRRL

TAPAIEVYTSESRFRDFLKTHGSELPIFVGFGLEASALEKLAHKHRNKGWFIVLGEYSEKAHEDFKFDER

HALVVLRGEDEVQDVYYGPFEGPDLVNFVKRNLPPLVTPLNIDSLKFLTEDGRPIVVGVLENNSTAEADA

FIKSMKAAAQANRDFVFASIVASQWPKFLRPFALGRKPVLPAVIIWDSKQYAKSDKTVDFGDQMESKVAD

LLQRFREKTITLLEVKGPTLYEKATENTTQMLQALILLFALFFQWIRKNREAAANLRREAPESEEPRSDQ

PRASEPSGVDEINADSNKED

**Phytozome v10.3: Phpat.004G055200.1**

>P.patensPDI-B2

MAASQSVRVFCFWAIALLLGFSSGRADDHVVEPAAAGRVLELTDDNFETEIAKHDHILVDFYAPWCGFCN

RLSPQLDIAAPVLVQGSRPIFLAKIDVEKHSKVGSKYKISAFPTLKYFIKGLPTDYTGPRKADGIITHLR

RLSAPSIEQLNSEAELHGFVKSIGTEIPLFIGFGLKATDLEEFANKYRSKAWFAVMETFSEEIMEDFNFD

KGPALVVTRGEHGEKTTFYGPFEAEDVTEFVLLNLLPLVSHMTPETLRSVREDGRPVVVAVLESESAPED

KEFIKKLKMAAPAYRKFVFAYVVAPQWPEFLRPFWIRKETKLPTVFVWTDDTFVVSNKSDAFIGSSVETE

LYNLLQMYQDNVLKRHKMRTPSLWERATANLPLTVGYGFLCLGILILLLKGWDTRAHQEVREEHSDATGG

SSAEGRGSRGDKKED

**NCBI Reference Sequence: XP_007225650.1**

>P.persicaPDI-B

MEKRDCSGVVYSSSSSWAVDGKVLELDDSNFDSAISALDLVLVDFYAPWCGHCKRLSPQLDEAAPLLADL

KQPIVIAKLNADKFTSLARKYEIDAYPTLKLFMHGVPIEYNGPRKADSLVRYLKKFAAPDVSILDSDSAI

SDFVQAAGTYFPIYIGFGLNESLISKLAIKYKKKAWFSVAKDFSEDVMVLYDFDKLPALASLHPTYNEHN

IFYGPYEEEFLEEFIRHSLFPLAMPINYETLKLLNDDERKIVLTIVEDEDEEKSKKLIKILKSAASANRD

FVFGYVGIKQWEDFADTFGANKKTRLPKMVVWDRMEEYFTVNGSESIDEEDQASQVSQFLEGYKEGRIIK

ERIGGPSFMGFMSSFIGIGTVYIIGFVVVVMMLIRSINKGDDEHPAVATGDQIDHASSEAENREHKSGEK

ED

**NCBI Reference Sequence: XP_002325550.2**

>P.trichocarpaPDI-B1

MKTRRSPLILLNTTPLLLLVLLSSSIISSAESTTPPEKINTVLELDESNFDSTISTYDYVFVDFYAPWCG

HCKRLAPELDVAAPILAELKKPIVIAKVNADKYTRLARKHEVDGFPTLKIYMHGVPTDYYGPRKAELLVC

FLRKFVAPDVTILNSDSAIREFVEEAGTHFPIFIGFGLNETVMSNLAIKYKKKAWFSVASDFSDDVMVQY

DFDKIPALVSIHPSYNDHTVFYGPFEEEFMEEFITQNSLPLAVPINSETLKVLKDDQRKIVLTILEDDSE

EKSQNLIKILKAAASANRDLVFGFVGVKQWEEFTETFGANKETKLPKMIVWDGDEEYLSVIGSESIEEED

QGSQISQFLAGYRGGRTERNRVSGPSLLGYISSLIGIRTVYIIVFLVAMLMFIQHISKEEPLRVGTRDQA

EPATSSKAESSEYRPEDKQD

**NCBI Reference Sequence: XP_006376276.1**

>P.trichocarpaPDI-B2

MKTTRSSLILILSIIISATESTRADKINTVLELDESNFDSTIAAYDYVFVDFYAPWCTHCKRLAPELDVA

APILAELKKPIVIAKVNADKYTRLARKHEVDGYPTLKIYMHGVPTEYYGPRKAELLVRFLRKFVAPDVVV

LNSDSAIREFVEEAGTHFPIFIGFGLNETLISNLAIKYKKKAWFSVASDFSDDVMVQYDFDKIPTVVSIH

PSYDDHSIFYGPFEEEFLEEFIEQNFLPLAVPINYDTLKVLKDDQRKIVLTILEDESEEKSQKLIKTLKA

AASANRNLVFGYVGVKQWAEFAETFGAKGTKLPKMIVWDGGEEYLSVIGSESIEEEDQGSQISQFLAGYR

EGKTERNRISGPSLMGYLNSLIGVRTVYIIVFLVAMLILIRHISKEEPLTVGTGDQVEHATSSEAESSDY

RPGDKQD

**NCBI Reference Sequence: XP_007143637.1**

>P.vulgarisPDI-B

MRMRVSVVLSVVLLIQFWPGEAETFSVDGRVLILDESNFDSAIASFDNILVDFYAPWCGHCKRLAPELDA

AAPVLATLNKPIVIAKVDADKHTRLGKKYDVSAYPTILLFNHGIPTEYRGPRKADLLVRYLKKFAASDVS

ILDSDSAVNTFVAEAGTFFPVFIGFGLNNMLIEKFGIKYKKQAWFSVAKDFSEDLMVLYDFDKIPALVSL

NPQYSERNTFYGPFEDEFLEDFVRQNLIPLVVPVSYETLKLVKADGRKIVLTIVEDEDEERSRELTKLLK

AAASANRDLVFGYVGVKQMEEFAENFDISTKLPKMVVWDKSDDYLSVVDSESIDGEDQATKISKFLEGYR

EGRTINKTFSGPSLMQFIHRSFDIRMVYIIVFVVAVLILIQTFSKDDEYHPVPNQVQEDDPSSSVSEAES

KEYKPGDKED

**NCBI Reference Sequence: XP_002447854.1**

>S.bicolorPDI-B1

MATRVLPPALLSLILLPLLLLSARDTVAAGEDFPRDGRVIDLDESNFEAALGAIDFLFVDFYAPWCGHCK

RLAPELDEAAPVLAGLSEPIVVAKVNADKYRKLGSKYGVDGFPTLMLFIHGVPIEYTGSRKADQLVRNLK

KFVSPDVSILESDSAIKTFVENAGTSFPMFLGFGVNDSLIAEYGRKYKKRAWFAVAKEFSEDIMVAYEFD

KVPALVAIHPKYKEQSLFYGPFEENFLEDFVRQSLLPLVVPINTETLKMLNDDQRKVVLTILEDDSDENS

TQLVKILRSAANANRDLVFGYVGIKQWDEFVETFDVSKSSQLPKLLVWDRNEEYELVDGSERLEEGDQAS

QISQFLEGYRAGRTTKKKISGPSFMGFLNSLVSLSSLYILIFVIALLVVMVYFAGQDDTPQPRRIHEE

**NCBI Reference Sequence: XP_002453968.1**

>S.bicolorPDI-B2

MAMALRRRLLLLLPLLFLVVLVQRPHNCVASGGGGGEPAEFEIPRDGSVLELDESNFEAAVRAAEFLFVD

FYAPWCGHCKRLAPQLDEAAVVLAGLSTPVVVAKVNADKYRKLGSKYGVDGFPTLMLFDHGVPSEYTGSR

KADLLVENLKKLVAPDVSVLESDSSIKGFVEAAGINFPLFIGFGMDESLIVEYGAKYKKKAWFSTAKDFS

EDMMVVYDFDKFPALVSVNPKYNEQSVFYDPFEVRNFSSLMVTPIVVLDTGTFLEDFIRQSLLPVTVPVD

RETVKLLKDDGRKVVLTILEDESDENSPQLIKVLRSAANANHDLVFGYVGVKQWEEFSETFDVKVPQLPK

IIVWDTKEEYEVVEGSESLREGDYGSQVSRFLEGYREGRTIKKKVGRGSPTLLGLNAIYILIFLVAVLVV

LMYFSAQGEEDHQPRRGRAHED

**NCBI Reference Sequence: XP_004250404.1**

>S.lycopersicumPDI-B1

MVPFISISIFTFGFLLLFRCSSSSAEQQQFAIDGKVLELDESNFEAAISTFDYMFVDFYAPWCGHCKRLS

PELDKASANLAVLKQPIVIAKVDADKYSRLASKYEIDGFPTLKIFMHGVPTDYYGPRKADLLVRFLKKFV

APDVSVLNSDSAISEFIEEAGKNFPIFIGFGLNESVISHLAVKYKKSAWFSVAKDFSDTTMEFYDFDKVP

ALVTLHLSYNEQSIFYGPFEEKFLEDYIKQSLLPLVLPINQDTLKSLKDDKRKIVLTIVEDEDDERSKRL

VKLLKAAASANRDLVFAFVGFKQWQDFAESFEVSKKIKLPKMIVWDGDVEYFSVIGSDSVEDEDQGSQIT

RFLKGYRDGSVIQKHIISDDYKAFRNSMFLIGALILVLVVILVAMMMQAVKEEPSREQVDHPGSSTSLSE

AREALRSGDKEEKID

**NCBI Reference Sequence: XP_004241830.1**

>S.lycopersicumPDI-B2

MMKNMSFVILLLVLVSRPFITAAESEQQKQQLGIDSGKVLELDESNFDAAISSFDYILVDFYAPWCGHCK

RLAPQLDKAASILADLKKPISIAKIDADKYKRVGSKYGIDGYPTLKIFMHGVPTEYYGPRKADLLVRFLK

KFVAADVAILNSDSAISEFVEAAGTSFPIFIGFGLNESAISHFAVKYKKRAWFSVAKDFSDKTMEFYDFD

KVPALVARHPNYDEQSIFYGPFEENFVEDYIKQSLLPLTLPITEETLRLLKDDERKVILTILEDETDDRS

KKLLKLLKAAASANRDFVFVFVGFKQWQGFAESFDVSKKTKLPKMVVWDGDEEYFSVVGSESVEDEDQGS

QITHFIQGYKDGNIIQKRISSGSFMGFINSMIGLGTVTIIVFVVAVVMIIQSLKEEPLTVGTRDEGDHPS

SSTSQTEARQPLRSGEKQDKED

**NCBI Reference Sequence: XP_002966260.1**

>S.moellendorffiiPDI-B

MLWAILSLLLLVAAEHDEGRLCRRGAVVELDGSNFDAAISECRNILVDFYAPWCSHCNALSPQLDEAASQ

LASEPEELTLAKINVDKFTAIASRYKINEYPTLKLFVDGIHTDYRGPHKAELMVAHLRRMLAPPLSTLQS

PSAVKQFVERAGDKLPVFVGFGVEVSTLAELAQGHRLRAWFATVDQEGSASELDLLSSDYGLTVLPALLV

QHSSMNEQAVFHGPFQGEGLASFVRHNLLPPVTTLTYDNLELVKADGRPVVLAIVTGAGVFNHMKELARE

HPEMLFALLNSSSPLADIFYASKVLVWDGKTYFYTRLHAEVSENEVEMGGQISALVEDFKNNKVKRSIIK

QPSFMEQLMGFIGQNVLYIVLFFVTIVVFLQSMDWAQPGAARH

**NCBI Reference Sequence: XP_006361368.1**

>S.tuberosumPDI-B1

MVPLRSISIFTFGFLLLFLCSSLAEQQQFAVDGKVLELDESNFEAAISTFDYMFVDFYAPWCGHCKRLSP

ELDKASVNLAVLKQPVVIAKVDADKYSRLASKYEIDGFPTLKIFMHGVPTDYYGPRKADLLVRFLKKFVA

PDVSVLNSDSAISEFIEEAGKNFPIFIGFGLNESVISHLAVKYKKSAWFSVAKDFSDKTMEFYDFDKVPA

LVALHLTYNEQSIFYGPFEEKFLEDYIKQSLLPLVLSINQDTLKSLKDDKRKIVLTIVEDEDDERSKGLV

KLLKAAASANRDLVFAFVGFKQWQDFAESFEVSKKIKLPKMIVWDGDVEYFSVIGSDSVEDEDQGSQITR

FLKGYREGSVIQKHIISDDYKAFRNSMFLIGALILVLVVILVSMMMQSVKEEPSREQVDHPGSSTSLSEA

RVALRSGDKEDKID

**NCBI Reference Sequence: XP_006353621.1**

>S.tuberosumPDI-B2

MMKNNTMSFLFIFLLLVLVSWPFITAAETEQQKQQFAVESGKVLELDESNFDAAISNFDYILVDFYAPWC

GHCKRLAPQLDKAASILADLKQPISIAKIDADKYKRVGSKYGIDGYPTLKIFMHGVPTEYYGPRKADLLV

RFLKKFVAADVAILNSDSAISEFVEAAGTSFPIFIGFGLNESAISHFAVKYKKRAWFSVAKDFSDKTMEF

YDFDKVPALVARHPNYDEQSIFYGPFEEKFVEDYIKQSLLPLTLPITEENLRLLKDDERKVILTILEDES

DDRSKKLVKLLKAAASANHDFVFVFVGFKQWQGFAESFEVSKKTKLPKMVVWDGDEEYFSVVGSDSIEDE

DQGSQITQFIQGYKDGNIIQKRISSGSFMGFINSMIGIGTVTIIVFVVAVVMIIQSLKEEPVTVGTRDEG

DHPSSSTSQAEARQPLRSGEKQDKED

**NCBI Reference Sequence: XP_007020002.1**

>T.cacaoPDI-B1

MRLLMILLLLLCIFRSGICSTADQFKVDGKVLELDESNFDSAISSFDYILVDFYAPWCGHCKRLSPQLDE

AAPVLAGLKEPIVIAKVNADKYTRLARKHDVDAYPTLKLFMHGVSMEYFGPRKAELLVQYLKKFVAPDVS

VLSSDSAISDFVEAAGSFFPIYIGFGLNETVTSNLAVKYKKRAWFSVAKDFSDDAMVLYDFDKVPALVAL

HPSYKQQSIFYGPFEDEFLGDFIKQNLLPLVVPLNHETLKLLKDEERKIVLTITADENEDQSQNLIKVLK

AAASANRDLVFGYVGVQQWEDFADKFGVDKKTKLPKMIVWNGDEEYFSVIGIESLDKEDQGSQISRFLEG

YREGRTERKTVKGPSFMGFINSLIGIRTVYIVVFIVAVIMLIQSIGKDDEPLGVGRRDEVDHAESSEAES

SQYGPEKKED

**NCBI Reference Sequence: XP_007020007.1**

>T.cacaoPDI-B2

MRLLTILCSLVLCLLKPGISATAPQFKVDGKVLELEESNFDSAISSFDYILVDFYAPWCGHCKRLSPQLD

EAAPVLAGLKEPIVIAKLNADKFTSLARKYEIGGYPTLKLFMHGVPVDYYGPRKADLLVRFLKKFVAPDV

SILGSDSAINDFVEAAGTYFPIYIGFGLNETVISNLAVKYKKKAWFSVAKDFSEDVMVLYDFDKVPALVV

LHPSYNQQTVFYGPFEDEFLGDFIKQNFLPLVVPMKHETLKLLKDDKRKIVLTIMEDENEEKSQKLIKLL

KAAASANRDLVFGYVGVKQWEEFADTFDGAEKTNFPKMIIWNGDEEYFSVIGSESLDDEDQGSQISRFLE

GYREGRLEKKRIKGPSFMDFINSLIGIRTVYIIVFLVAMLILIRSLGKDDEPLRVGTRDEVDHAESSTAE

SSGYRPGEKED

**NCBI Reference Sequence: XP_002269656.1**

>V.viniferaPDI-B

MEGRLSVMGVGVATLSIFALLFSSVSSHDFPTDGTVLELHDSNFDSAISAFDFILVDFYAPWCGHCKRLA

PELDAAAPVLASLKEPIVIAKVNADKFTRLAVKYDIDGFPTLKLFIRGVPMDYYGPRKADLLVRFLKKFV

APDVSVLVSDSAISSFVEAAGTHFPIYIGFGLNESMISNLAIKYKKKAWFSVAKDFSEDVMVAYDFDKVP

ALVSLHPSYNEHSVFYGPFDGEFLEDFMKQTLFPLVLPINYDTLKLLKDDDRKIFLTFVDDELDEKSKKL

INLLKAAASANRDLVFGFVGIKQWGEFADSFGANKKTKLPKMVVWDGDNQYFEVIGSESFDEIDQGSQIT

RFIEGYKEGRTVEKKIGGPSLIGYINSLIGIRTVYIIVFVVAVMMIIQSINKEEPLTIGSGEDQTDQAWS

SSLTDESREEYRPRDKED

**NCBI Reference Sequence: NP_001105761.1**

>Z.maysPDI-B1

MATRVLPPALLSFILLLLLSLSARDTVAAGEDFPRDGRVIDLDDSNFEAALGAIDFLFVDFYAPWCGHCK

RLAPELDEAAPVLSGLSEPIVVAKVNADKYRKLGSKYGVDGFPTLMLFIHGVPIEYTGSRKADQLVRNLK

KFVSPDVSILESDSAIKNFVENAGISFPIFLGFGVNDSLIAEYGRKYKKRAWFAVAKDFSEDIMVAYEFD

KVPALVAIHPKYKEQSLFYGPFEENFLEDFVRQSLLPLVVPINTETLKMLNDDQRKVVLTILEDDSDENS

TQLVKILRSAANANRDLVFGYVGIKQWDGFVETFDVSKSSQLPKLLVWDRDEEYELVDGSERLEEGDQAS

QISQFLEGYRAGRTTKKKITGPSFMGFLNSLVSLNSLYILIFVIALLFVMVYFAGQDDTPQPRRIHEE

**NCBI Reference Sequence: NP_001105803.1**

>Z.maysPDI-B2

MAARVLPPPPLPLVLLLLLLPLSARDTVAAGEDFPRDGRVIDLDESNFEAALGVIDFLFVDFYAPWCGHC

KRLAPELDEAAPMLAGLSEPIVVAKVNADKYRKLGSKYGVDGFPTLMLFIHGVPIEYTGSRKADQLVRNL

KKFVAPDVSILESDSAIKNFVENAGTSFPMFLGFGVNDSLIAEYGRKYKKRAWFAVAKDFSEDVMVAYEF

DKVPALVAIHPKYKEQSLFYGPFEENFLEDFVRQSLLPLVVPINTETIKMLNDDQRKVVLTILEDDSDEN

STQLVKILRSAASANRDLVFGYVGIKQWDEFVETFDVSKSSQLPKLLVWDRNEEYELVDGSERLEEGTDQ

ASQISQFLEGYRAGRTTKKKISGPSFMGFLNSLVSLTSLYILIFVIALLFVMVYFAGQDDTPQPRRIHEE

**NCBI Reference Sequence: XP_008643659.1**

>Z.maysPDI-B3

MAMALRRLLLPLLLLVLLGLRPQSCVASGGGGGEPAEFEIPRDGSVLELDESNFEAAVRAAEFLFVDFYA

PWCGHCKRLAPQLDEAAAVLAGLSTPVLVAKVNADKYKKLGSKYGVDGFPTLMFFDHGVPSEYTGSRKAD

VLVENLKKLVAPDVSVLESDSSINGFVQAAGINFPLFIGFGMDESLIVEYGAKYKKKAWFSTAKDFSEDV

MVVYDFDKVPALVSVNPKYNEQSVFYGPFEGTFLEDFIRQSLLPATVPINRETVKLLKDDGRKVVLTILE

DESDESSLQLIKVLRSAANANHDLVFGYVGVKQWEEFTETFDVKVSQLPKIVVWDTKEEYEVVEGSESFI

EGDYGSQVSRFLEGYREGRTTKKKVGRGSPTLLGLNAVYILVLLVAVLVVLMYFSAQGEEDHQPRRAHED
